# Disease Severity Staging System for *NOTCH3*-Associated Small Vessel Disease, Including CADASIL

**DOI:** 10.1001/jamaneurol.2024.4487

**Published:** 2024-11-29

**Authors:** Gido Gravesteijn, Julie W. Rutten, Minne N. Cerfontaine, Remco J. Hack, Yi-Chu Liao, Amy A. Jolly, Stéphanie Guey, Shao-Lun Hsu, Jae-young Park, Yun Yuan, Anna Kopczak, Nicola Rifino, Sam J. Neilson, Anna Poggesi, Md Manjurul Islam Shourav, Satoshi Saito, Hiroyuki Ishiyama, Ana Domínguez Mayoral, Renata Nogueira, Elena Muiño, Pia Andersen, Nicola De Stefano, Gustavo Santo, Nontapat Sukhonpanich, Francesco Mele, Ashley Park, Jung Seok Lee, Mar Rodríguez-Girondo, Sebastiaan J. J. Vonk, Amy Brodtmann, Anne Börjesson-Hanson, Leonardo Pantoni, Israel Fernández-Cadenas, Ana Rita Silva, Vinícus V. A. Montanaro, Rajesh N. Kalaria, Diego Lopergolo, Masafumi Ihara, James F. Meschia, Keith W. Muir, Anna Bersano, Francesca Pescini, Marco Duering, Jay Chol Choi, Chen Ling, Hyunjin Kim, Hugh S. Markus, Hugues Chabriat, Yi-Chung Lee, Saskia A. J. Lesnik Oberstein

**Affiliations:** 1 Department of Clinical Genetics, Leiden University Medical Center, Leiden, the Netherlands; 2 Department of Neurology, Taipei Veterans General Hospital, Taipei, Taiwan; 3 Department of Neurology, Brain Research Center, and Institute of Clinical Medicine, National Yang Ming Chiao Tung University, Taipei, Taiwan; 4 Stroke Research Group, Department of Clinical Neurosciences, Cambridge Biomedical Campus, University of Cambridge, Cambridge, United Kingdom; 5 Centre NeuroVasculaire Translationnel and Centre de Référence des Maladies Vasculaires Rares du Cerveau et de L’Oeil, Lariboisière Hospital and Université Paris-Cité, Paris, France; 6 Institut National de la Santé et de la Recherche Médicale, Unit 1141 NeuroDiderot, Paris, France; 7 Department of Neurology, Asan Medical Center, University of Ulsan College of Medicine, Seoul, South Korea; 8 Department of Neurology, Peking University First Hospital, Beijing, China; 9 Institute for Stroke and Dementia Research, University Hospital of Ludwig-Maximilians-University Munich, Munich, Germany; 10 Cerebrovascular Unit, Fondazione IRCCS Istituto Neurologico Carlo Besta, Milan, Italy; 11 Centre for Stroke & Brain Imaging, University of Glasgow, Glasgow, United Kingdom; 12 Stroke Unit, Careggi University Hospital, Florence, Italy; 13 Dipartimento di Neuroscienze, Psicologia, Area del Farmaco e Salute del Bambino, University of Florence, Florence, Italy; 14 Department of Neurology, Mayo Clinic, Jacksonville, Florida; 15 Department of Neurology, National Cerebral and Cardiovascular Center, Suita, Japan; 16 Unidad de Enfermedades Neurovasculares Infrecuentes, Hospital Virgen Macarena, Sevilla, Spain; 17 Department of Neurology, Hospital SARAH Kubitschek, Rio de Janeiro, Brazil; 18 Stroke Pharmacogenomics and Genetics Group, Institut de Recerca Sant Pau, Hospital de la Santa Creu i Sant Pau, Barcelona, Spain; 19 Theme Inflammation and Aging, Karolinska University Hospital, Stockholm, Sweden; 20 Department of Neurobiology, Care Sciences and Society, Karolinska Institutet, Stockholm, Sweden; 21 Department of Medicine, Surgery and Neurosciences, University of Siena, Siena, Italy; 22 Department of Neurology, University Hospital of Coimbra, Unidade Local de Saúde de Coimbra, Coimbra, Portugal; 23 Department of Medicine, Faculty of Medicine Siriraj Hospital, Mahidol University, Bangkok, Thailand; 24 Neurology and Stroke Unit, Luigi Sacco University Hospital, Milan, Italy; 25 Department of Neurology, Royal Melbourne Hospital, Melbourne, Australia; 26 College of Medicine, Jeju National University, Jeju, South Korea; 27 Department of Biomedical Data Sciences, Leiden University Medical Center, Leiden, the Netherlands; 28 Software developer in personal capacity, Weesp, the Netherlands; 29 School of Translational Medicine, Monash University, Melbourne, Australia; 30 Neuroscience Research Center, Department of Biomedical and Clinical Sciences, University of Milan, Milan, Italy; 31 Center for Research in Neuropsychology and Cognitive Behavioral Interventions, University of Coimbra, Coimbra, Portugal; 32 Department of Neurology, Hospital SARAH Kubitschek, Brasília, Brazil; 33 Translational and Clinical Research Institute, Newcastle University, Newcastle, United Kingdom; 34 School of Cardiovascular & Metabolic Health, University of Glasgow, Glasgow, United Kingdom; 35 Medical Image Analysis Center and Translational Imaging in Neurology, Department of Biomedical Engineering, University of Basel, Basel, Switzerland; 36 Center for Intelligent Drug Systems and Smart Bio-Devices, National Yang Ming Chiao Tung University, Hsinchu, Taiwan

## Abstract

**Importance:**

Typical cysteine-altering *NOTCH3* (*NOTCH3*^cys^) variants are highly prevalent (approximately 1 in 300 individuals) and are associated with a broad spectrum of small vessel disease (SVD), ranging from early-onset stroke and dementia (cerebral autosomal dominant arteriopathy with subcortical infarcts and leukoencephalopathy [CADASIL]) to nonpenetrance. A staging system that captures the full *NOTCH3*-SVD severity spectrum is needed and currently lacking.

**Objective:**

To design a simple disease severity staging system that captures the broad clinicoradiological *NOTCH3*-SVD severity spectrum.

**Design, Setting, and Participants:**

A cohort study was performed in which the *NOTCH3*-SVD severity staging system was developed using a discovery cohort (2019-2020) and validated in independent international CADASIL cohorts (1999-2023) and the UK Biobank. Clinical and imaging data were collected from participants originating from 23 international CADASIL cohorts and from the UK Biobank. Eligibility criteria were presence of a *NOTCH3*^cys^ variant, availability of brain magnetic resonance imaging, and modified Rankin Scale score. The discovery cohort consisted of 195 *NOTCH3*^cys^-positive cases from families with CADASIL; the validation set included 1713 *NOTCH3*^cys^-positive cases from 15 countries. The UK Biobank cohort consisted of 101 *NOTCH3*^cys^-positive individuals. Data from 2-year (2019-2023) and 18-year (1999-2017) follow-up studies were also analyzed. Data analysis was performed from July 2023 to August 2024.

**Main Outcomes and Measures:**

Percentage of cases following the sequence of events of the *NOTCH3*-SVD stages, and the association between the stages and ischemic stroke, intracerebral hemorrhage, global cognition, processing speed, brain volume, brain microstructural damage, and serum neurofilament light chain (NfL) level.

**Results:**

The *NOTCH3*-SVD staging system encompasses 9 disease stages or substages, ranging from stage 0 (premanifest stage) to stage 4B (end stage). Of all 1908 cases, which included 195 in the discovery cohort (mean [SD] age, 52.4 [12.2] years) and 1713 in the validation cohorts (mean [SD] age, 53.1 [13.0] years), 1789 (94%) followed the sequence of events defined by the *NOTCH3*-SVD staging system. The *NOTCH3*-SVD stages were associated with neuroimaging outcomes in the *NOTCH3*^cys^-positive cases in the CADASIL cohorts and in the UK Biobank and with cognitive outcomes and serum NfL level in cases from the CADASIL cohorts. The *NOTCH3*-SVD staging system captured disease progression and was associated with 18-year survival.

**Conclusions and Relevance:**

The *NOTCH3*-SVD staging system captures the full disease spectrum, from asymptomatic individuals with a *NOTCH3*^cys^ variant to patients with end-stage disease. The *NOTCH3*-SVD staging system is a simple but effective tool for uniform disease staging in the clinic and in research.

## Key Points

**Question:**

Can a simple but effective disease severity staging system be designed to capture the broad *NOTCH3*-associated small vessel disease (*NOTCH3*-SVD) severity spectrum?

**Findings:**

In this cohort study, a *NOTCH3*-SVD staging system captured the broad *NOTCH3*-SVD severity spectrum, followed the natural *NOTCH3*-SVD course, and was associated with neuroimaging and clinical outcome measures.

**Meaning:**

The findings suggest the *NOTCH3*-SVD staging system will help to better harmonize *NOTCH3*-SVD and cerebral autosomal dominant arteriopathy with subcortical infarcts and leukoencephalopathy (CADASIL) cohort studies and registries; may improve individualized disease counseling, monitoring, and clinical management; and may facilitate patient stratification in clinical trials.

## Teaser

This cohort study designed a disease severity staging system to capture the broad clinicoradiological severity spectrum of *NOTCH3*-associated small vessel disease, including cerebral autosomal dominant arteriopathy with subcortical infarcts and leukoencephalopathy (CADASIL).

## Introduction

Cerebral small vessel disease (SVD) is a major contributor to stroke, disability, and cognitive decline worldwide.^1^ The most frequent monogenic cause of SVD is cerebral autosomal dominant arteriopathy with subcortical infarcts and leukoencephalopathy (CADASIL). The prevalence of CADASIL has been reported to be minimally 4 cases per 100 000 individuals,^2^ but cysteine-altering *NOTCH3* (*NOTCH3*^cys^) variants, classically associated with CADASIL, have recently been shown to occur at a high frequency in population databases worldwide (2-10 cases per 1000 individuals).^3,4,5^ Community-dwelling individuals with *NOTCH3*^cys^ variants have an increased lifetime risk for stroke and vascular dementia, although with a later onset than in patients with diagnosed CADASIL.^6,7,8^ This has led to the insight that the disease spectrum associated with *NOTCH3*^cys^ variants is extremely broad, ranging from severely affected individuals in families with mid-adulthood–onset stroke and dementia to individuals with a milder, seemingly sporadic, late-onset SVD.^9,10,11,12^ The position of the *NOTCH3*^cys^ variant is a key determinant of *NOTCH3*-associated SVD (*NOTCH3*-SVD) severity, above cardiovascular risk factors and sex.^13,14,15,16^ *NOTCH3*^cys^ variants can be classified into high, moderate, or low risk for developing severe mid-adulthood–onset *NOTCH3*-SVD, also known as CADASIL.^17^

Although there is a large variability in age at onset and rate of disease progression, the order in which the cardinal features of *NOTCH3*-SVD occur is fairly uniform. The initial and ubiquitous feature is white matter hyperintensities (WMHs) on brain magnetic resonance imaging, preceding symptoms by many years.^18,19^ WMHs are progressive and, in more advanced disease stages, accompanied by lacunes, cerebral microbleeds, and brain atrophy. Cardinal clinical symptoms include migraine with aura, recurrent strokes, cognitive impairment, apathy, and depression.^19,20,21^

Various neuroimaging and clinical outcome measures are used to describe *NOTCH3*-SVD severity at the individual and cohort levels,^16,21,22^ but, to our knowledge, there is currently no validated disease staging system that covers the full disease severity spectrum associated with *NOTCH3*^cys^ variants, from asymptomatic individuals to patients with end-stage disease. We aimed to design a simple disease staging system that does not require advanced data processing, to enable a broad implementation in diverse clinical and research settings, facilitating comparisons of cohorts across study sites, disease monitoring, biomarker discovery, and clinical trial stratification.

Herein, we propose a *NOTCH3*-SVD severity staging system that was designed and validated using clinical and neuroimaging data of 1908 cases with a *NOTCH3*^cys^ variant from CADASIL cohorts from 15 countries, and 101 individuals with a *NOTCH3*^cys^ variant from the UK Biobank.

## Methods

### Ethics

All procedures performed in studies involving human participants were in accordance with the 1964 Declaration of Helsinki and its later amendments or comparable ethical standards. Ethical approval was obtained for all cohorts (eAppendix 1 in the Supplement). Written informed consent was obtained from participants in all cohorts, except for cohorts with a waiver from the local institutional review board owing to the use of deidentified data (cohorts from Seoul, Korea; Sevilla, Spain; Newcastle, United Kingdom; Glasgow, United Kingdom; Melbourne, Australia; Jacksonville, Florida; and Brasília, Brazil). The Strengthening the Reporting of Observational Studies in Epidemiology (STROBE) reporting guidelines were followed. Data analysis was conducted from July 2023 to August 2024.

### Selection of Neuroimaging and Clinical Features in the Discovery Cohort

To enable the capture of the full *NOTCH3*-SVD severity spectrum, from premanifest to end-stage disease, we considered both CADASIL neuroimaging and clinical features for the staging system. A list of candidate features was compiled based on expert opinion and literature review (eAppendix 2 and eTable 1 in Supplement 1). Clinical and neuroimaging features were considered for inclusion if (1) they are readily and uniformly obtained in standard clinical (neurological) practice, (2) the feature occurs in at least one-third of patients with CADASIL in the course of the disease, and (3) the risk of developing the feature increases with age, as a proxy for disease progression. For criterion 3, we assessed how the proportion of patients with this feature increased across 5 age categories (ages 25-34, 35-44, 45-54, 55-64, and ≥65 years) within a discovery cohort, which included comprehensive clinical and neuroimaging data of 195 patients with CADASIL and family members with premanifest disease (eFigure 1 in Supplement 1).^15,17^ In this discovery cohort, a sensitivity analysis to assess the impact of microbleeds on the *NOTCH3*-SVD staging system was performed (eTable 2 in Supplement 1).

### Validation Cohorts

Twenty-two CADASIL cohorts of cases from families with CADASIL were used to validate the applicability of the *NOTCH3*-SVD staging system as well as its association with disease markers not included in the staging system itself. The inclusion criteria were (1) documented *NOTCH3*^cys^ variant or positive findings on skin biopsy,^23,24^ and (2) availability of Fazekas deep white matter score,^25^ lacune count according to Standards for Reporting Vascular Changes on Neuroimaging 1 (STRIVE-1) or STRIVE-2 criteria,^26^ and modified Rankin Scale (mRS) score. A *NOTCH3*^cys^ variant was defined as a *NOTCH3* variant leading to a cysteine amino acid change in one of the 34 epidermal growth factor–like repeat domains of the NOTCH3 protein^27^ or as one of several well-documented causative *NOTCH3* cysteine-sparing variants (p.Arg61Trp, p.Arg75Pro, p.Asp80Gly, p.Arg213Lys [n = 53]).^28^ Description of all cohorts can be found in eTable 3 and eAppendix 3 in Supplement 1. Individuals with a *NOTCH3*^cys^ variant in the UK Biobank with brain magnetic resonance imaging results available were separately analyzed as they were ascertained by a genotype-first approach rather than via a CADASIL diagnosis or positive CADASIL family history; as such, they represent the mildest end of the *NOTCH3*-SVD severity spectrum.^9^

### Association Between the *NOTCH3*-SVD Staging System and Clinical and Neuroimaging Measures

The association between the *NOTCH3*-SVD staging system and 7 disease outcome measures not included in the staging system itself was assessed in the discovery cohort and in 22 validation cohorts for which at least 1 of these measures was (made) available for this study (eTable 3 and eAppendix 3 in Supplement 1). The following outcome measures were selected based on their known association with CADASIL disease severity^21,22^: ischemic stroke, intracerebral hemorrhage (ICH), global cognition (Mini-Mental State Examination, Montreal Cognitive Assessment, or Cambridge Cognitive Examination), processing speed (Trail Making Test parts A and B or executive function components of the Brief Memory and Executive Test^29^ or Symbol Digit Modalities Test), brain parenchymal fraction (BPF),^30^ peak width of skeletonized mean diffusivity (PSMD),^31^ and serum neurofilament light chain (NfL) level.^32,33^

### Statistical Analysis

Nonnormally distributed data were transformed to obtain normal distribution: global cognition scores were reflected (31 minus score) and cube rooted; PSMD and NfL level were natural log transformed. Processing speed was expressed as *z* scores per cohort. Standardized values were used in statistical analyses and illustrations. The difference in sex and stage distribution between cohorts was assessed using χ^2^ tests. The associations between the *NOTCH3*-SVD staging system (as categorical independent variable) and BPF, PSMD, global cognition, processing speed, and serum NfL level were determined in the discovery cohort using linear regression analyses and then tested in the validation cohorts using linear mixed models. Cohort was included as a random effect in these analyses to take cohort differences as well as missing data into account. The association of the *NOTCH3*-SVD staging system with ischemic stroke and ICH was assessed using logistic regression. Bonferroni correction for multiple testing was performed for the association between the *NOTCH3*-SVD stages and the outcome measures. Cox regression and log-rank analyses were used to estimate the association between the *NOTCH3*-SVD stages (stages 0-1B, 2A-2B, and 3A-4A were combined to have similar group sizes) and survival. All analyses were performed with and without adjusting for age and sex. Ethnicity could not be taken into account due to collinearity with cohort as a random factor. Probabilities of fulfilling stage criteria were estimated using logistic regression analyses, with and without stratifying for *NOTCH3* variant risk category. Analyses were performed in R version 4.3.0 statistical software (R Foundation). Statistical tests were 2-sided with the threshold for statistical significance set as *P* < .05.

## Results

### Design of the *NOTCH3*-SVD Staging System

**Figure 1.**
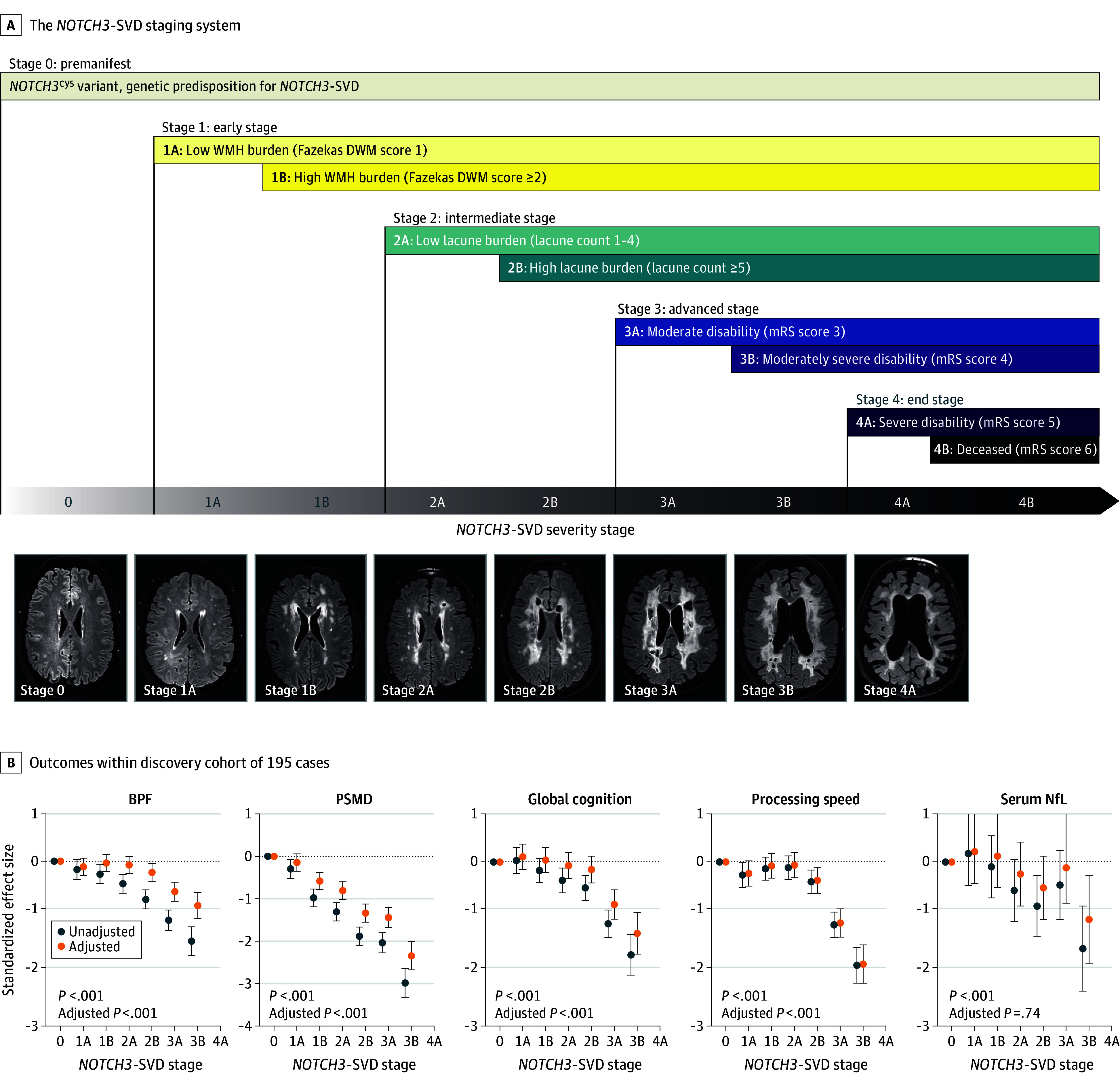
The *NOTCH3*–Small Vessel Disease (SVD) Staging System A, The *NOTCH3*-SVD staging system comprises 5 main stages, of which stages 1 to 4 are subdivided into 2 substages. Cases are assigned to the highest stage for which they fulfill the criterion, even if cases do not fulfill all criteria of the lower stages (see also eFigure 2 in Supplement 1). The *NOTCH3*-SVD staging system can be applied to any individual with a cysteine-altering *NOTCH3* (*NOTCH3*^cys^) variant and to individuals with well-documented, causative cysteine-sparing *NOTCH3* variants. Per stage, a representative T2-weighted fluid-attenuated inversion recovery magnetic resonance image is shown. DWM indicates deep white matter; mRS, modified Rankin Scale; WMH, white matter hyperintensity. B, Within the discovery cohort of 195 cases, the *NOTCH3*-SVD staging system was associated with outcomes of brain parenchymal fraction (BPF), peak width of skeletonized mean diffusivity (PSMD), global cognition, processing speed, and serum neurofilament light chain (NfL) level. Blue dots indicate unadjusted standardized effect sizes; yellow dots, standardized effect sizes adjusted for age and sex; error bars, standard error. *NOTCH3*-SVD stage 4A was not included as there were no cases in this stage in the discovery cohort. Unadjusted *P* values and *P* values adjusted for age and sex are for comparison between the *NOTCH3*-SVD staging system and outcomes.

Twenty of the 24 identified candidate clinical and neuroimaging features did not fulfill the criteria for inclusion in the *NOTCH3*-SVD staging system. A comprehensive description of the design, including selection of features and thresholds, can be found in eAppendix 2 in Supplement 1. The *NOTCH3*-SVD staging system encompasses 5 disease stages ranging from 0 to 4, with stages 1 to 4 each being divided into 2 substages for a total of 9 substages. The features fulfilling the criteria and selected for inclusion were Fazekas deep white matter (DWM) score, lacune count, and mRS score. The Fazekas DWM score captures early disease stages (stage 1A, Fazekas DWM score 1; stage 1B, Fazekas DWM score ≥2), the lacune count captures intermediate disease stages (stage 2A, count >0; stage 2B, count ≥5), and the mRS score captures advanced disease stages (stage 3A, mRS score 3; stage 3B, mRS score 4) and end-stage disease (stage 4A, mRS score 5; stage 4B, mRS score 6). Stage 0 was included for individuals with a *NOTCH3*^cys^ variant but with no features of SVD (Fazekas DWM score 0, no lacunes) (Figure 1A).

### Association Between the *NOTCH3*-SVD Staging System and Other Disease Outcome Measures

Among the 195 cases (mean [SD] age, 52.4 [12.2] years) in the discovery cohort, the clinical and neuroimaging features were in line with the sequence of events as defined in the *NOTCH3*-SVD staging system in 186 cases (95%) (eFigure 2 and eTable 3 in Supplement). The *NOTCH3*-SVD stages were significantly associated with BPF (*F*_7,188_ = 108; *P* < .001; adjusted *F*_6,186_ = 6.1; adjusted *P*< .001; for effect sizes, see eTable 4 in Supplement 1), PSMD (*F*_7,171_ = 40.3; *P* < .001; adjusted *F*_6,169_ = 19.6; adjusted *P* < .001), global cognition (*F*_7,175_ = 9.8; *P* < .001; adjusted *F*_6,173_ = 6.3; adjusted *P* < .001), and processing speed (*F*_7,188_ = 28.2; *P* < .001; adjusted *F*_6,186_ = 13.6; adjusted *P* < .001). *NOTCH3*-SVD stages were also associated with serum NfL levels, but this was not significant after correction for age and sex (*F*_7,58_ = 6.7; *P* < .001; adjusted *F*_6,56_ = 1.8; adjusted *P* = .74) (Figure 1B). The prevalence of transient ischemic attacks, gait disturbance, cognitive impairment, apathy, seizures, and microbleeds also increased across the stages. Migraine with aura and depression were present in an almost equal proportion of patients in each of the stages (eFigure 3 in Supplement 1). The *NOTCH3*-SVD staging system had better resolution and performance than a previously published grading system for CADASIL (eFigure 4 and eTable 5 in Supplement 1).^34^

### Validation in 22 International CADASIL Cohorts

**Figure 2.**
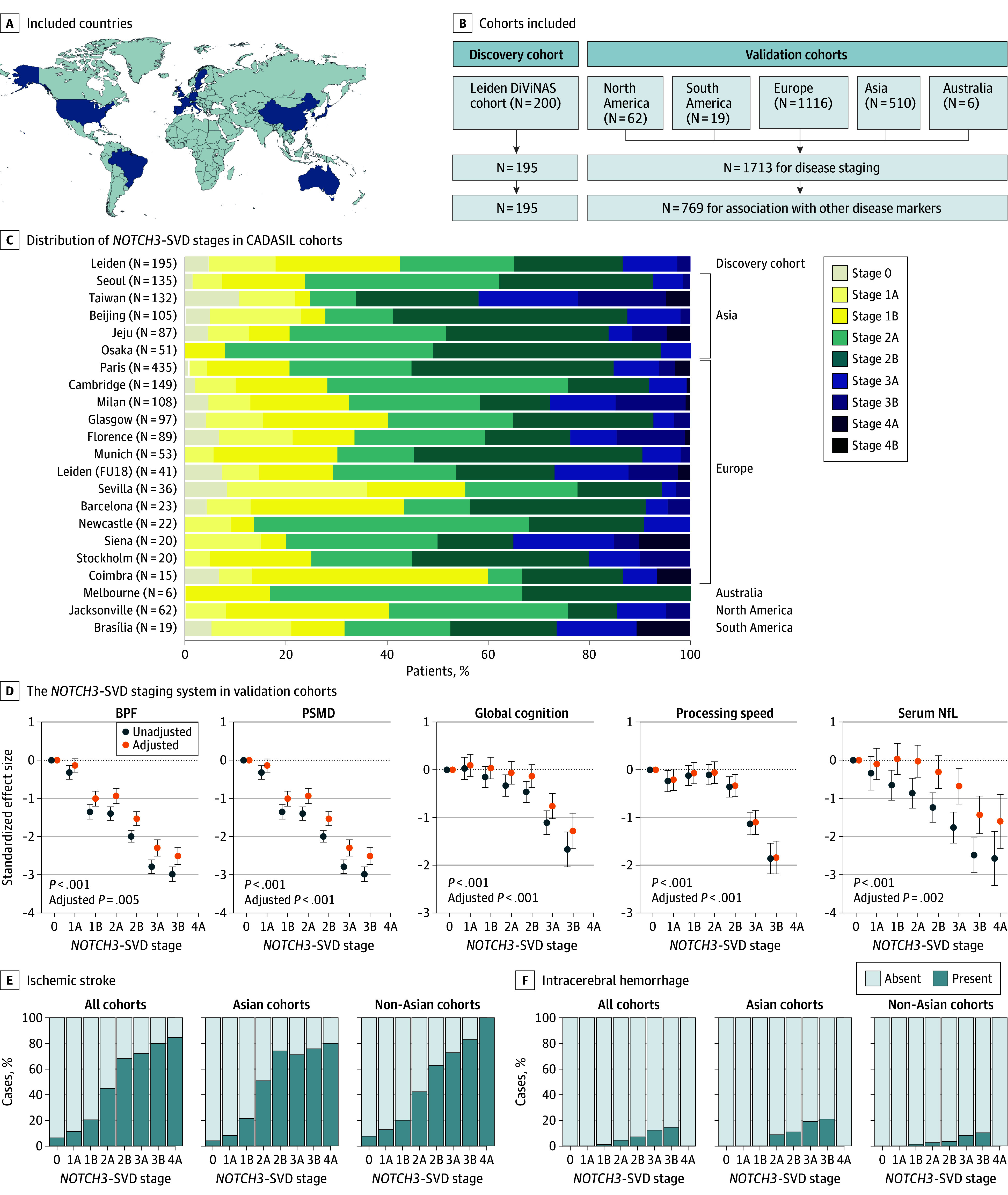
Validation of the *NOTCH3*–Small Vessel Disease (SVD) Staging System in Independent Cohorts A and B, The *NOTCH3*-SVD staging system was validated in 1713 cases from 22 cohorts with cerebral autosomal dominant arteriopathy with subcortical infarcts and leukoencephalopathy (CADASIL) from 15 countries. DiViNAS indicates Disease Variability in *NOTCH3*-Associated Small Vessel Disease. C, The *NOTCH3*-SVD staging system revealed differences in the distribution of disease stages between the cohorts (χ^2^_154_ = 542; *P* < .001). FU18 indicates the baseline cohort of the 18-year follow-up study. D, Like in the discovery cohort, the *NOTCH3*-SVD staging system was associated with brain parenchymal fraction (BPF), peak width of skeletonized mean diffusivity (PSMD), global cognition, processing speed, and serum neurofilament light chain (NfL) levels in the validation cohorts. Blue dots indicate unadjusted standardized effect sizes; yellow dots, standardized effect sizes adjusted for age and sex; error bars, standard error. Adjusted *P* values are adjusted for age and sex. E and F, Proportion of patients with a history of ischemic stroke (E) and intracerebral hemorrhage (F) per *NOTCH3*-SVD stage (*P* < .001 for all). Intracerebral hemorrhage was more prevalent in Asian cohorts than in non-Asian cohorts.

The *NOTCH3*-SVD staging system was tested in 1713 cases (mean [SD] age, 53.1 [13.0] years) from 22 independent CADASIL cohorts from Europe, Asia, South America, Australia, and the United States (Figure 2A-C; eTable 3 in Supplement 1). Similar to the discovery cohort, 1603 cases (94%) had an ordered sequence of events (eFigure 2 in Supplement 1). The proportion of cases in each stage differed between the cohorts (χ^2^_154_ = 542; *P* < .001).

In line with the results from the discovery cohort, *NOTCH3*-SVD stages were associated with neuroimaging measures (BPF: χ^2^_8_ = 96; *P* < .001; adjusted χ^2^_7_ = 25; adjusted *P*= .005; PSMD: χ^2^_7_ = 228; *P* < .001; adjusted χ^2^_6_ = 153; adjusted *P*< .001), clinical measures (global cognition: χ^2^_8_ = 239; *P* < .001; adjusted χ^2^_7_ = 155; adjusted *P* < .001; processing speed: χ^2^_8_ = 219; *P* < .001; adjusted χ^2^_7_ = 163; adjusted *P* < .001), and serum NfL level (χ^2^_8_ = 55; *P* < .001; adjusted χ^2^_7_ = 28; adjusted *P* = .002) in the 769 cases from the validation cohorts for which the association with disease markers could be determined (Figure 2D; eTable 4 and eFigure 5 in Supplement 1). The prevalence of ischemic stroke increased across the *NOTCH3*-SVD stages (χ^2^_7_ = 334; *P* < .001) (Figure 2E). There was a higher prevalence of ICH in Asian cohorts compared with European cohorts (Figure 2F), in line with previous reports.^35^ In Asian cohorts, ICH occurred exclusively in cases in *NOTCH3*-SVD stage 2A or higher. On average, male patients were in higher disease stages than female patients (χ^2^_7_ = 70; *P* < .001) (eFigure 6 in Supplement 1).

### Validation in Individuals With a *NOTCH3*^cys^ Variant in the UK Biobank

**Figure 3.**
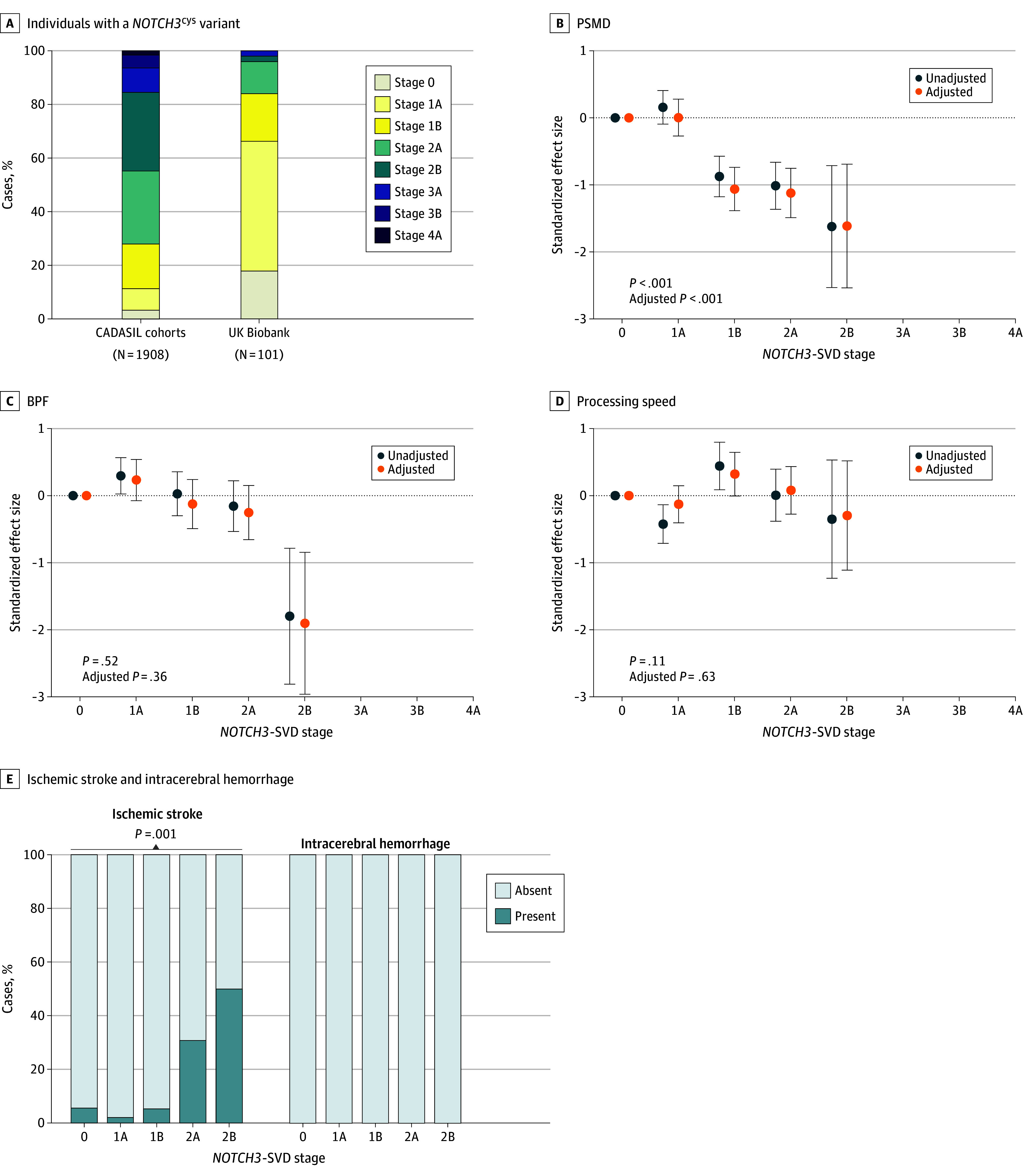
*NOTCH3*–Small Vessel Disease (SVD) Stages in Individuals With a Cysteine-Altering *NOTCH3* (*NOTCH3*^cys^) Variant in the UK Biobank A, Individuals with a *NOTCH3*^cys^ variant in the UK Biobank were at lower *NOTCH3*-SVD stages compared with cases from cohorts with cerebral autosomal dominant arteriopathy with subcortical infarcts and leukoencephalopathy (CADASIL) (χ^2^_7_ = 241; *P* < .001). B-D, *NOTCH3*-SVD stages in individuals with a *NOTCH3*^cys^ variant in the UK Biobank were associated with peak width of skeletonized mean diffusivity (PSMD) (B) but not with brain parenchymal fraction (BPF) (C) or processing speed (D). Blue dots indicate unadjusted standardized effect sizes; yellow dots, standardized effect sizes adjusted for age and sex; error bars, standard error. E, Proportion of individuals with a history of ischemic stroke (χ^2^_4_ = 17.6; *P* = .001) or intracerebral hemorrhage per *NOTCH3*-SVD stage in the UK Biobank.

To test the *NOTCH3*-SVD staging system in individuals at the mildest end of the *NOTCH3*-SVD severity spectrum, the staging system was applied to 101 individuals with a *NOTCH3*^cys^ variant in the UK Biobank. Of these, 86 (85%) were in stage 0 to 1B (compared with 471 of 1713 cases [28%] in the CADASIL cohorts) (Figure 3A). *NOTCH3*-SVD stage was significantly associated with PSMD (*F*_5,87_ = 5.9; *P* < .001; adjusted *F*_4,85_ = 5.2; adjusted *P* < .001) and ischemic stroke prevalence (χ^2^_4_ = 17.6; *P* = .001) but not with BPF (*F*_5,91_ = 1.3; *P* = .52; adjusted *F*_4,89_ = 1.3; adjusted *P* = .36), processing speed (*F*_5,58_ =1.9; *P* = .11; adjusted *F*_4,56_ = 0.6; adjusted *P* = .63), or ICH prevalence (Figure 3B-E).

### *NOTCH3*-SVD Staging System, Disease Progression, and *NOTCH3* Variant Risk Category

**Figure 4.**
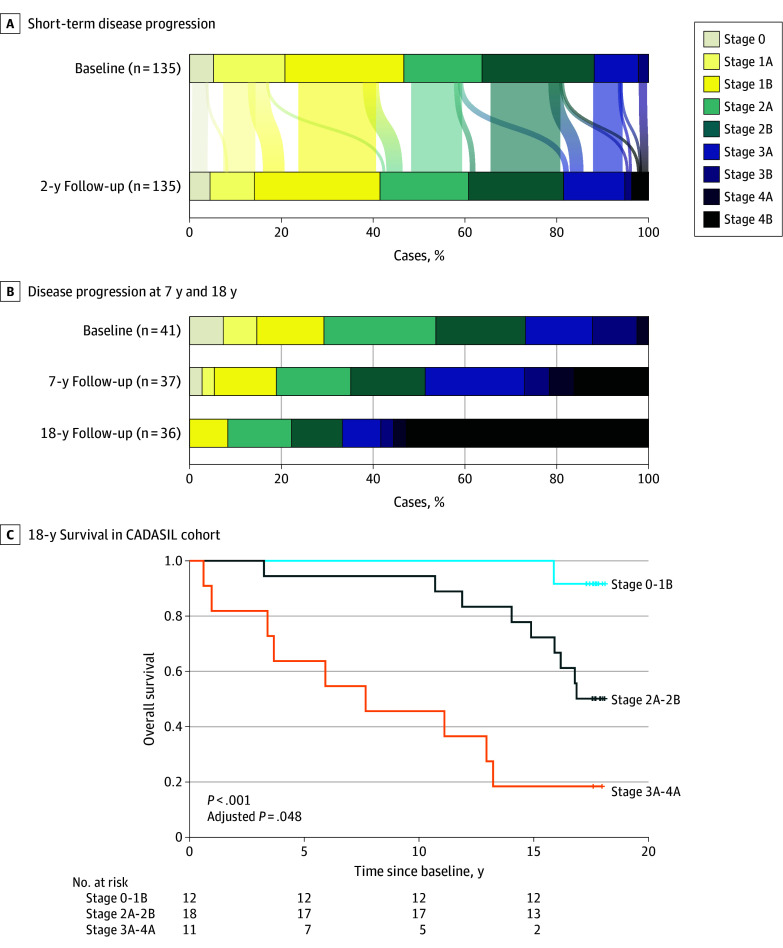
*NOTCH3*–Small Vessel Disease (SVD) Staging System Captures Short-, Intermediate-, and Long-Term Disease Progression A, The *NOTCH3*-SVD staging system was used to illustrate short-term disease progression, revealing that the majority of cases (76%) remain within the same stage over the course of 2 years, while 17% progress 1 stage and only 7% progress 2 stages or more. B, Disease progression at 7 and 18 years according to the *NOTCH3*-SVD staging system. After 7 years, approximately half of the cases progressed 1 stage, while after 18 years, 85% of the cases progressed at least 1 stage. C, Survival at 18 years was associated with *NOTCH3*-SVD stages at baseline before and after adjustment for age and sex in cohorts with cerebral autosomal dominant arteriopathy with subcortical infarcts and leukoencephalopathy (CADASIL).

In a 2-year follow-up study of 135 cases from a CADASIL cohort, 103 (76%) remained in the same *NOTCH3*-SVD stage, while 23 (17%) progressed to the next stage, irrespective of *NOTCH3*-SVD stage at baseline (Figure 4A). Seven cases (7%) progressed more than 1 stage.

In a midterm (7-year) and long-term (18-year) follow-up study of 41 Dutch patients with CADASIL, 8 of 9 patients (88%) who were in stage 3A or higher at baseline had progressed at least 1 stage at 7-year and 18-year follow-up. Of the 12 cases who were in stage 0 to 1B at baseline, 7 (58%) progressed at least 1 stage at 7-year follow-up and 10 (83%) progressed at least 1 stage at 18-year follow-up (Figure 4B). After 18 years, among 41 cases, 6 (15%) were still in the same stage as they were at baseline, 4 (10%) progressed 1 stage, and 26 (63%) progressed 2 or more stages. Higher *NOTCH3*-SVD stages at baseline were associated with lower 18-year survival (log-rank χ^2^_2_ = 17.9; *P* < .001; Cox regression χ^2^_2_ = 6.1; adjusted *P* = .048) (Figure 4C).

**Figure 5.**
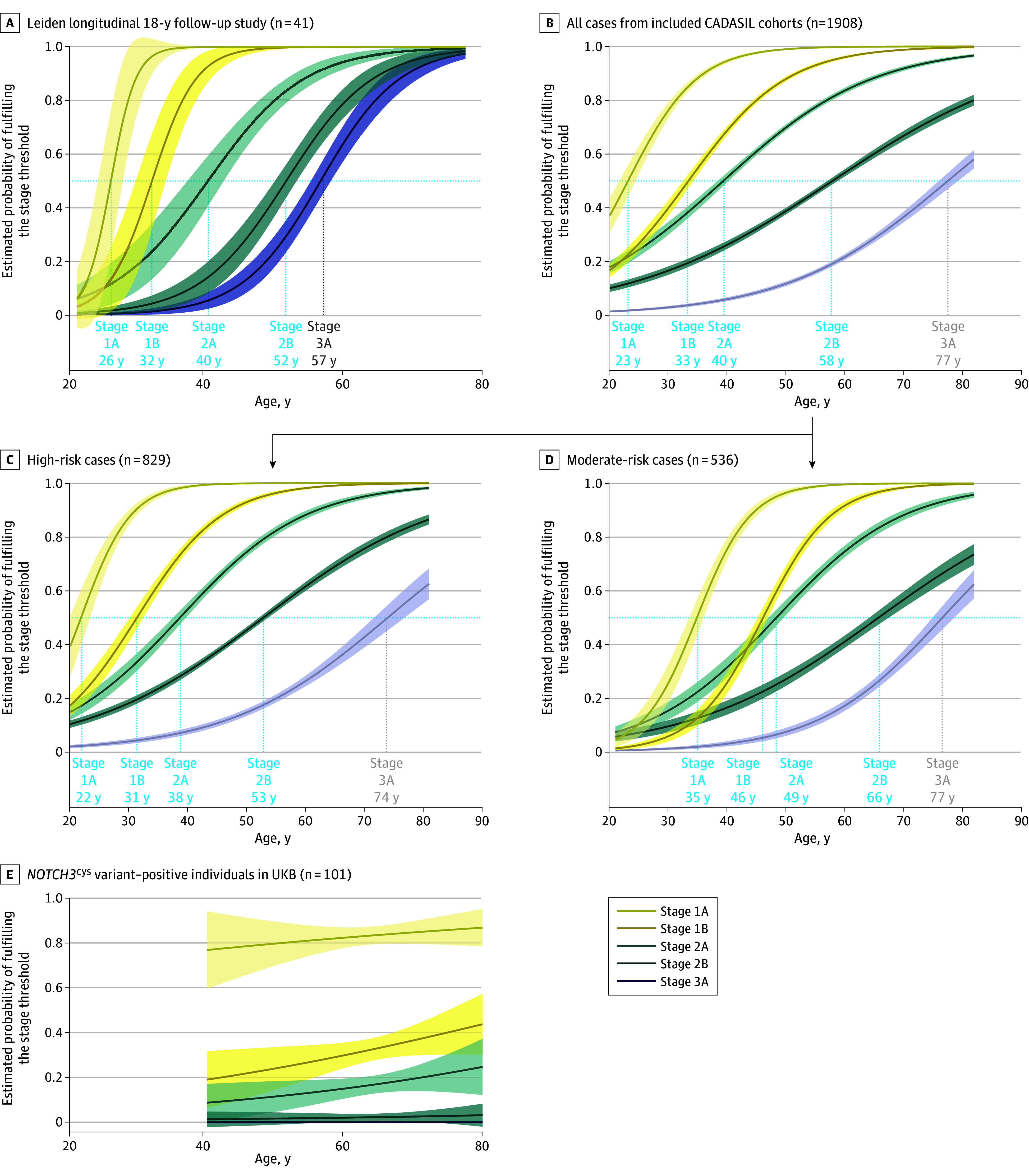
Age Estimation Models in Cases With a Cysteine-Altering *NOTCH3* (*NOTCH3*^cys^) Variant for Each Stage of the *NOTCH3*–Small Vessel Disease (SVD) Staging System A, The estimated probability of fulfilling the criteria for stages 1A to 3A was based on longitudinal data of 41 cases from a validation cohort who were followed up for 18 years. B, The estimated probability of fulfilling the criteria for stages 1A to 3A was based on cross-sectional data of all included cases from cohorts with cerebral autosomal dominant arteriopathy with subcortical infarcts and leukoencephalopathy (CADASIL). C and D, The estimated probability of fulfilling the criteria for stages 1A to 3A was stratified by *NOTCH3* variant risk category. As expected, the estimated probability of cases with a high-risk *NOTCH3*^cys^ variant and of a certain age to be in a certain stage (C) was higher compared with cases with a moderate-risk *NOTCH3* variant at the same age (D). The probability for being classified into stage 3A is underestimated due to survival bias. A-D, The age at which 50% of the individuals were estimated to be in a specific stage or higher is depicted. E, Estimated probabilities for *NOTCH3*^cys^-positive individuals in the UK Biobank (UKB) show a much lower probability to fulfill the criteria for *NOTCH3*-SVD stages, even at older age.

Finally, we estimated the probability of cases fulfilling the criterion for a disease stage by age 40 years. In the 18-year follow-up study, all cases were estimated to meet the criterion for stage 1A by age 40 years, 50% were estimated to meet the criterion for stage 2A, and 7% to meet the criterion for stage 3A (Figure 5A). In a cross-sectional analysis of all 1908 cases, 95% were estimated to meet the criterion for stage 1A by age 40 years, 51% for stage 2A, and 6% for stage 3A (Figure 5B). In the UK Biobank, 77% of *NOTCH3* variant–positive individuals were estimated to meet the criterion for stage 1A by age 40 years, 9% for stage 2A, and only 1% for stage 2B (Figure 5E). Stratification based on *NOTCH3* variant risk category illustrated that cases with a high-risk *NOTCH3* variant had a higher estimated probability of meeting the criterion for a certain stage at a younger age than cases with a moderate-risk *NOTCH3* variant (Figure 5C and D).

## Discussion

The impetus for developing the *NOTCH3*-SVD staging system was the recently recognized broad SVD severity spectrum associated with *NOTCH3*^cys^ variants,^9,10^ the recent discovery of a high population frequency of these variants worldwide,^3,4,36^ and the lack of a system to uniformly define and communicate disease severity. We aimed to develop a staging system for *NOTCH3*-SVD severity that is easy to use in daily clinical practice and captures key *NOTCH3*-SVD clinicoradiological manifestations, from premanifest to end-stage disease. The *NOTCH3*-SVD staging system was designed using genetic, clinical, and neuroimaging data in a discovery cohort of 195 cases and was validated in 1713 cases from 22 CADASIL cohorts around the world. In addition, we included neuroimaging and clinical information from 101 *NOTCH3*^cys^ variant–positive individuals in the UK Biobank. In this way, the complete spectrum of *NOTCH3*-SVD severity as well as potential differences between ethnicities were taken into account.

The *NOTCH3*-SVD staging system encompasses 5 disease stages ranging from 0 to 4, with stages 1 to 4 each being divided into 2 substages, forming a total of 9 substages. The neuroimaging measures we included (Fazekas DWM score and lacune count) have been shown to be associated with relevant disease outcomes, such as apathy,^37^ and have the added benefit of allowing for discrimination of neuroimaging severity stages in individuals with clinically premanifest disease. Validity was demonstrated by showing that the staging system is associated with other SVD clinical and neuroimaging measures, such as stroke, microstructural white matter damage, and brain atrophy. The *NOTCH3*-SVD staging system captures disease progression, even in a short span of 2 years of follow-up, and is associated with long-term mortality. The *NOTCH3*-SVD stage can therefore serve as a starting point for individualized disease course modeling, in which *NOTCH3* variant risk category, ascertainment context, family history, age, and cardiovascular risk factor burden should also be taken into account.^13,14,15,16^ This is important, as a *NOTCH3*^cys^ variant can be ascertained in diverse situations, ranging from a young patient with stroke and cognitive impairment to an incidental next-generation sequencing finding in an asymptomatic individual. In any of these situations, the molecularly diagnosed individual can be assigned to one of the *NOTCH3*-SVD stages. The staging system also reflects the known difference in disease severity between *NOTCH3* variant risk categories,^17^ as we show that patients with high-risk variants are assigned to higher stages at younger ages. Individuals with a high-risk variant will likely progress through (almost) all stages in their lifetime, while individuals with a low-risk variant may progress no further than stage 1B. A major advantage of the *NOTCH3*-SVD staging system is that it can be easily implemented in clinical practice and will improve patient counseling, monitoring, management, and in the future possibly disease prediction. Future cohort studies will also benefit from the staging system, as it will ensure a uniform description of disease severity and facilitate patient stratification. An automated tool to stage (groups of) individuals with a *NOTCH3*^cys^ variant is freely available online.^38^

*NOTCH3*^cys^ variants occur at a high frequency in the general population worldwide, with an estimated average prevalence of 1 case in 300 individuals, with the highest frequency seen in Asian individuals (1 in 100).^3,4,5^ Stroke in patients with *NOTCH3*-associated SVD is almost exclusively ischemic small vessel stroke, but in Asian patients ICH is also relatively common and associated with prevalent variants in Asian patients (p.R544C and p.R75P).^35,39^ The *NOTCH3*-SVD staging system was also applicable to Asian patients despite these differences in ICH prevalence. The *NOTCH3*-SVD staging system might also be applicable to other genetic SVDs, such as *HTRA1*-associated SVD or even sporadic SVD.^40,41^

The *NOTCH3*-SVD staging system outperforms a previously published severity grading scale proposed for CADASIL,^34^ as it captures the full disease severity spectrum, discriminates between 9 disease stages, and was validated in a large sample of patients with CADASIL who were from diverse cohorts and ethnicities.

### Limitations

This study has several limitations. Although we could determine the *NOTCH3*-SVD stage in 1908 cases, a limitation is that we could perform the association analysis between the *NOTCH3*-SVD stages and other disease outcome measures for only a subset of cases (n = 769). The interrater agreement of the *NOTCH3*-SVD staging system was not assessed, as it would mainly be determined by the established interrater reliability of the Fazekas DWM score, lacune count, and mRS score.^16,42,43,44^ The accuracy of the age estimation models for patients at each stage of the *NOTCH3*-SVD staging system is constrained by survivor bias, resulting in an underestimation of the likelihood of being in an advanced disease stage. The longitudinal data we had available to improve these estimations was limited by either a relatively short follow-up period or a small sample size. The *NOTCH3*-SVD staging system was not designed to fulfill the criteria for a clinical scale and therefore cannot (yet) be used as an outcome measure for clinical studies or trials. The staging system also does not necessarily reflect disease burden experienced by a patient, as not all CADASIL symptoms were eligible for inclusion in the system, for example, migraine with aura, depression, and apathy, which can severely impact quality of life. Although we tried to include patients from all over the world, most are from Europe and East Asia, as so far CADASIL cohorts are not reported or are less frequently reported from other regions. However, the European cohorts do include patients of non-European extraction.

## Conclusions

We have designed an easy-to-implement staging system to uniformly assess *NOTCH3*-SVD severity stage in individuals with a *NOTCH3*^cys^ variant. Using validation in a large number of cases from multiple cohorts originating from numerous countries, representing all ages and disease stages, we show that the staging system is generalizable and reproducible. Widespread use of the *NOTCH3*-SVD staging system will contribute to better harmonization of cohort studies; may improve individualized disease counseling, monitoring, and management in the clinic; and may facilitate patient stratification in clinical trials.

## Notes

Supplement 1. **eAppendix 1.** Ethical Approval **eAppendix 2.** Design of the NOTCH3-SVD Staging System **eAppendix 3.** Cohort Information **eTable 1.** Selection of Features for the NOTCH3-SVD Staging System **eTable 2.** Microbleeds Do Not Improve NOTCH3-SVD Staging System **eTable 3.** Cohort Characteristics **eTable 4.** Effect Sizes for the Association Between the NOTCH3-SVD Stages and Age, Global Cognition, Processing Speed, BPF, PSMD, and NfL **eTable 5.** Statistical Comparison of the NOTCH3-SVD Staging System With the Previously Reported Anisetti System **eFigure 1.** CADASIL and NOTCH3-SVD Features by Age **eFigure 2.** Sequentiality of the NOTCH3-SVD Staging System in the Discovery and Validation Cohorts **eFigure 3.** Distribution of CADASIL Features per NOTCH3-SVD Stage **eFigure 4.** Graphical Comparison of the NOTCH3-SVD Staging System With the Previously Reported Anisetti System **eFigure 5.** Boxplots Showing the Association Between the NOTCH3-SVD Staging System and Age, Global Cognition, Processing Speed, BPF, PSMD and NfL per Cohort **eFigure 6.** Distribution of NOTCH3-SVD Stages by Sex

Supplement 2. Data Sharing Statement
