# Supplementary material for: Disease Severity Staging System for NOTCH3-Associated Small Vessel Disease, Including CADASIL
Source: JAMA Neurol. 2024 Nov 29;82(1):49–60. doi: 10.1001/jamaneurol.2024.4487 (PMC13340440; doi:10.1001/jamaneurol.2024.4487)
Supplement: Supplement 1. — eAppendix 1. Ethical Approval eAppendix 2. Design of the NOTCH3-SVD Staging System eAppendix 3. Cohort Information eTable 1. Selection of Features for the NOTCH3-SVD Staging System eTable 2. Microbleeds Do Not Improve NOTCH3-SVD Staging System eTable 3. Cohort Characteristics eTable 4. Effect Sizes for the Association Between the NOTCH3-SVD Stages and Age, Global Cognition, Processing Speed, BPF, PSMD, and NfL eTable 5. Statistical Comparison of the NOTCH3-SVD Staging System With the Previously Reported Anisetti System eFigure 1. CADASIL and NOTCH3-SVD Features by Age eFigure 2. Sequentiality of the NOTCH3-SVD Staging System in the Discovery and Validation Cohorts eFigure 3. Distribution of CADASIL Features per NOTCH3-SVD Stage eFigure 4. Graphical Comparison of the NOTCH3-SVD Staging System With the Previously Reported Anisetti System eFigure 5. Boxplots Showing the Association Between the NOTCH3-SVD Staging System and Age, Global Cognition, Processing Speed, BPF, PSMD and NfL per Cohort eFigure 6. Distribution of NOTCH3-SVD Stages by Sex [file jamaneurol-e244487-s001.pdf]

Supplemental Online Content

Gravesteijn G, Rutten JW, Cerfontaine MN, et al. Disease severity staging system for *NOTCH3*-associated small vessel disease, including CADASIL. *JAMA Neurol*. Published online November 29, 2024. doi:10.1001/jamaneurol.2024.4487

eAppendix 1. Ethical Approval

eAppendix 2. Design of the NOTCH3-SVD Staging System

eAppendix 3. Cohort Information

eTable 1. Selection of Features for the NOTCH3-SVD Staging System

eTable 2. Microbleeds Do Not Improve NOTCH3-SVD Staging System

eTable 3. Cohort Characteristics

eTable 4. Effect Sizes for the Association Between the NOTCH3-SVD Stages and Age, Global Cognition, Processing Speed, BPF, PSMD, and NfL

eTable 5. Statistical Comparison of the NOTCH3-SVD Staging System With the Previously Reported Anisetti System

eFigure 1. CADASIL and NOTCH3-SVD Features by Age

eFigure 2. Sequentiality of the NOTCH3-SVD Staging System in the Discovery and Validation Cohorts

eFigure 3. Distribution of CADASIL Features per NOTCH3-SVD Stage

eFigure 4. Graphical Comparison of the NOTCH3-SVD Staging System With the Previously Reported Anisetti System

eFigure 5. Boxplots Showing the Association Between the NOTCH3-SVD Staging System and Age, Global Cognition, Processing Speed, BPF, PSMD and NfL per Cohort

eFigure 6. Distribution of NOTCH3-SVD Stages by Sex

This supplemental material has been provided by the authors to give readers additional information about their work.

© 2024 Gravesteijn G et al. *JAMA Neurology.*

eAppendix 1: Ethical Approval

All procedures performed in studies involving human participants were in accordance with the 1964 Helsinki Declaration and its later amendments or comparable ethical standards. The Leiden DiViNAS study and FU18 study were approved by the Leiden-The Hague-Delft medical ethics committee (P18.164/P21.013 and P80.89/P17.170, respectively). The Cambridge cohort was recruited within the UK Cerebral Small Vessel Disease Study and was approved by the East of England–Cambridge Central Research Ethics Committee (16/EE/0118). The Munich cohort consisted of participants from two different prospective studies: VASCAMY and Zoom@SVDs. Both studies were approved by the Ethics Committeemedical ethics committee of the LMU Munich (project no. 158-13 for the VASCAMY study, and project no. 17-088 for the Zoom@SVDs study). The Taiwan cohort was recruited with approval by the institutional review board of Taipei Veterans General Hospital (TVGH IRB No. 2017-02-008A). The study resulting in the Jeju cohort was approved by the Institutional Review Board of Jeju National University Hospital, and all subjects gave written informed consent in accordance with the Declaration of Helsinki at the time of baseline examination. The Institutional Review Board of Asan Medical Center approved the study resulting in the Seoul cohort (approval number: 2023-1064). The cohort of patients from the IRCCS Besta Neurological Institute in Milan was recruited within the GENICA Study and was approved by the Lombardia 4 ethics committee (CET 50/23). The second Milan cohort (Sacco Hospital) was approved by the Comitato Etico Territoriale Lombardia 1 (CET 119-2024). The study resulting in the Sevilla cohort was approved by the local ethics committee under S1900024. The Swedish cohort (Stockholm cohort) was recruited within the study CADASIL in Sweden, which was approved by Swedish Ethical Review Authority (Drn 2019-03851). The Osaka cohort was approved by the National Cerebral and Cardiovascular Center Research Ethics Committee (R23013-3). The Newcastle cohort was approved by the local research ethics committee of the Newcastle upon Tyne Hospitals NHS Foundation Trust and the NHS Health Research Authority, North East - Newcastle & North Tyneside 1 Research Ethics Committee. For the Glasgow cohort, ethical approval for imaging was attained through the NHS Safe Haven (GSH/18/NE/007) with Caldicott approval for use of clinical data. For the Paris cohort, informed consent was obtained from each subject or from a close relative if necessary. The study was approved by an independent ethics committee (CEEI-IRB-17/388). The Coimbra cohort study was submitted and obtained approval from the Ethics Committee of the Faculty of Psychology and Educational Sciences of the University of Coimbra (CEDI7FPCEUC:78/12). The Florence cohort was recruited within the observational study on CADASIL patients approved by the local Ethics Committee (14381_oss). The Jacksonville cohort was evaluated through systematic review of the Mayo Clinic electronic medical record under the IRB protocol 23-01205. The Brazilian cohort was approved by the ethics committee of the SARAH Network of Rehabilitation Hospitals. Recruitment for the Beijing cohort has been conducted from April 2016 to August 2023 at Peking University First Hospital, and was approved by the Institutional Review Board and Ethics Committee at Peking University First Hospital. Informed consent was obtained from all participant in the following cohorts: Leiden DiViNAS, Leiden FU18, Cambridge, Munich, Taiwan, Jeju, Milan, Stockholm, Osaka, Barcelona, Paris, Siena, Coimbra, Florence, and Beijing. A waiver was granted by the institute review board or local ethics committee for the following cohorts: Seoul, Sevilla, Newcastle, Glasgow, Melbourne, Jacksonville and Brasilia.

© 2024 Gravesteijn G et al. *JAMA Neurology.*

eAppendix 2: Design of the NOTCH3-SVD Staging System

The list of candidate clinical and neuroimaing features to be included in the staging system was compiled based on expert opinion and literature review. PubMed was queried using the following search terms: ("CADASIL" OR "NOTCH3 small vessel disease") AND (("multicenter" OR "single center" OR "patient cohort" OR "cohort study" OR "disease prediction" OR "disease staging") OR (“cohort”)). We identified 24 candidate clinical and neuroimaging features for potential inclusion in the NOTCH3-SVD staging system (eTable1).

Eleven clinical and neuroimaging features did not fulfill the first selection criterion of being readily and uniformly obtainable obtained in standard clinical (neurological) practice. For example, the neuroimaging features PSMD, normalized WMH volume and normalized lacune volume were excluded, as these are not readily obtained as part of standard clinical practice. The clinical feature cognitive impairment was excluded as this is not uniformly diagnosed, and the onset is often difficult to define. History of TIA was also excluded, as it is known that discriminating between atypical migraine aura, and TIA in CADASIL can be challenging. Stroke was also excluded, as in the discovery cohort we found that there were patients who had numerous larger lacunes on MRI, but no reported history of stroke.

Three candidate features (seizures, CADASIL-associated encephalopathy, and intracerebral hemorrhage) did not fulfill criterion 2, as they did not occur in more than a third of patients in the discovery cohort (eFigure 1). A threshold of one-third was chosen to ensure that only the cardinal clinical and neuroimaging features would be included in the staging system. We did not choose a higher cutoff as this would exclude features that are commonly known to be present in end stage disease (example: a high mRS score), but are underrepresented in ambulant clinical CADASIL cohorts such as the DiViNAS discovery cohort.

Migraine with aura and depression were excluded based on criterion 3, as their prevalence did not increase across the age categories in the discovery cohort (eFigure 1A). Disability measured using mRS was the clinical feature that most clearly increased across the five age categories (eFigure 1B).

Seven features fulfilled the three criteria: four clinical features and three neuroimaging features. mRS was one of these clinical features, and was able to also capture the other three clinical features: mRS score 4 was used as a proxy for significant gait disturbances, mRS score 5 was used as proxy for being bedridden, and mRS 6 reflects death (eTable 1). The three remaining candidate neuroimaging SVD markers (Fazekas dwm, lacune count, microbleed count) all increased with age (eFigure 1C). Lacune count increased after age 35, while microbleed count only increased after age 45, suggesting that lacune count is a more sensitive measure in young patients. Therefore lacune count was selected as feature for inclusion in the staging system and not microbleed count. A sensitivity analysis showed that not including microbleeds in the staging system does not adversely affect the performance of the staging system (eTable 2).

Finally, the features selected for inclusion in the NOTCH3-SVD staging system were Fazekas dwm, lacune count and mRS. Fazekas dwm score was used to capture early disease stages, and a subdivision was included to capture differences in white matter lesion load in early (pre-manifest) disease stages (stage 1A Fazekas dwm 1 and 1B Fazekas dwm ≥2). Lacune count was used for intermediate disease stages; the subdivision in 2 substages was based on the median lacune count of individuals in stage 2 in the discovery cohort, leading to a subdivision into stage 2A (lacune count 1-4) and 2B (lacune count ≥5). In a sensitivity analysis, adding microbleed count to the staging system did not result in a better performing model (eTable 2).

© 2024 Gravesteijn G et al. *JAMA Neurology.*

eAppendix 3: Cohort Information

**Cohort 1**	**Leiden cohort (DiViNAS cohort)**
References	doi.org/10.1161/STROKEAHA.122.039325
Used as	Discovery cohort<br>Validation cohort for 2-year changes in NOTCH3-SVD stages (n=136)
N	195
Setting	Patients and family members with a *NOTCH3*^{cys} variant were recruited (in 2020-2021) from the Dutch expertise center for CADASIL at the department of Clinical Genetics.
In- and exclusion criteria	Included were individuals with a *NOTCH3*^{cys} variant, who were aged ≥ 18 years, able to participate in a single research day at the hospital including neuroimaging and a cognitive test battery. Excluded were individuals who were not able to participate due to severe disability, or had contraindications for neuroimaging.
Global cognition	Measured by MOCA (available for 182 cases). For analyses, values were reflected (31-score) and then cube rooted and expressed as standardized score.
Executive function	Measured by Trail Making Test part A and part B. Expressed as average of age-, sex- and education-normalized *z*-scores.
BPF	Available for all cases.
PSMD	PSMD values (available for 178 cases) were natural log transformed.
Blood NfL	NfL values (available for 65 cases) were natural log transformed.
Remarks	Supported by the Netherlands Organisation for Health Research and Development (ZonMW 91717325) and the Netherlands Brain Foundation (HA2016-02-03).<br>Please note that out of the 195 patients who participated at baseline, data was available for 136 patients at 2-year follow-up due to lost-to-follow-up (doi.org/10.1212/WNL.0000000000209310).

**Cohort 2**	**Leiden cohort (18-year follow-up [FU18]-cohort)**
References	doi.org/10.5853/jos.2020.04112
Used as	Validation cohort<br>Validation cohort for 7- and 18-year changes in NOTCH3-SVD Stages
N	41
Setting	Patients and family members with a *NOTCH3*^{cys} variant were recruited (in 1999-2000) from the Dutch expertise center for CADASIL at the department of Clinical Genetics. Patients were followed-up after 7 years (n=37) and 18-years (n=36). As a consequence, 18-year survival data of this cohort was available.
In- and exclusion criteria	Included were patients and family members with a *NOTCH3*^{cys} variant, who were aged ≥ 18 years, able to participate in a single research day at the hospital including neuroimaging and a cognitive test battery. Excluded were individuals who were not able to participate at a single research day at the hospital.
Global cognition	Measured by CAMCOG. For analyses, values were reflected (31-score) and then cube rooted and expressed as standardized score.
Executive function	Measured by TMT-A and TMT-B (available for 40 cases). Expressed as average of age-, sex- and education-normalized *z*-scores.
BPF	Available for 40/41 cases.
PSMD	Not available.
Blood NfL	NfL values (available for 40 cases) were natural log transformed.
Remarks	Supported by the Netherlands Brain Foundation (HA2016-02-03; BG2015-2).

**Cohort 3**	**Cambridge cohort**
References	doi.org/10.1212/WNL.0000000000200607
Used as	Validation cohort
N	149
Setting	Patients were recruited to the UK Familial Cerebral Small Vessel Disease Study (FSVD) at 6 UK neurologic sites, or after being referred to the Cambridge CADASIL Clinic, and prospectively characterized.

© 2024 Gravesteijn G et al. *JAMA Neurology.*

In- and exclusion criteria	Included were adult individual with a *NOTCH3*[cys] variant. Excluded were individuals with no known or suspected form of monogenic SVD, unwillingness to consent and those <18 years old.
Global cognition	Measured by MOCA (available for 139 cases). For analyses, values were reflected (31-score) and then cube rooted and expressed as standardized score.
Executive function	BMET, part executive function (available for 143 cases). For analyses, values were standardized.
BPF	Available for 146 cases.
PSMD	Not available.
Blood NfL	Not available.
Remarks	Supported by the Cambridge BHF Centre of research Excellence (RE/18/1/34212), the Cambridge University Trust Biomedical Research Centre, and the British Heart Foundation (RG/F/22/110052).

**Cohort 4**	**Munich cohort**
References	doi.org/10.5853/jos.2017.02565
Used as	Validation cohort
N	53
Setting	Prospectively collected data of patients with CADASIL who underwent full characterization on one day or over two consecutive days.
In- and exclusion criteria	Included were adults with a *NOTCH3*[cys] variant or ultrastructural analysis of skin biopsy showing granular osmiophilic material. Excluded were individuals with diabetes mellitus, other known neurologic of psychiatric disease, and individuals with clinically apparent stroke in last 3 months.
Global cognition	Measured by MMSE. For analyses, values were reflected (31-score) and then cube rooted and expressed as standardized score.
Executive function	Measured by TMT-A and TMT-B. Expressed as average of age-, sex- and education-normalized z-scores.
BPF	Available for all cases.
PSMD	Available for all cases.
Blood NfL	Available for all cases, natural log transformed.
Remarks	Supported by the Vascular Dementia Research Foundation.

**Cohort 5**	**Taiwanese cohort**
References	doi.org/10.1212/WNL.0000000000008700
Used as	Validation cohort
N	132
Setting	Both symptomatic CADASIL patients with stroke/TIA or cognitive decline, as well as their family members without clinical manifestations from a neurology outpatient clinic/ward were enrolled.
In- and exclusion criteria	Included were individuals with a *NOTCH3*[cys] variant or with a well-documented cysteine-sparing *NOTCH3* variant, regardless of their clinical symptoms or severity of leukoencephalopathy. Excluded were individuals who were unable to undergo brain MRI.
Global cognition	Measured by MMSE (available for 97 cases). For analyses, values were reflected (31-score) and then cube rooted and expressed as standardized score.
Executive function	Measured by TMT-A and TMT-B (available for 101 cases). Expressed as average of age-, sex-normalized z-scores.
BPF	Available for 101 cases.
PSMD	Available for 101 cases. PSMD values were natural log transformed.
Blood NfL	Not available.
Remarks	

**Cohort 6**	**Jeju cohort**
References	doi.org/10.3389/fnagi.2020.591879
Used as	Validation cohort
N	87
Setting	Neurology department.

© 2024 Gravesteijn G et al. *JAMA Neurology.*

In- and exclusion criteria	Included were patients identified at neurology department with a *NOTCH3*^{cys} variant or with a well-documented cysteine-sparing *NOTCH3* variant, who were symptomatic. In addition, presymptomatic individuals with a *NOTCH3*^{cys} variant or with a well-documented cysteine-sparing *NOTCH3* variant, who were identified through family screening were included. Excluded were patients who were unable to undergo brain MRI.
Global cognition	Measured by MMSE (available for 86 cases). For analyses, values were reflected (31-score) and then cube rooted and expressed as standardized score.
Executive function	Not available.
BPF	Available for 79 cases.
PSMD	Not available.
Blood NfL	Not available.
Remarks	-

**Cohort 7**	**Seoul cohort**
References	doi.org/10.1371/journal.pone.0234797
Used as	Validation cohort
N	135
Setting	Patients from a neurology outpatient clinic who were retrospectively reviewed.
In- and exclusion criteria	Included were CADASIL patients with a *NOTCH3*^{cys} variant or with a well-documented cysteine-sparing *NOTCH3* variant, who visited Asan medical center, Seoul, Korea, between 2003/01/01 – 2022/12/31. Excluded were patients who were unable to complete neuroimaging.
Global cognition	Measured by MMSE (available for 61 cases). For analyses, values were reflected (31-score) and then cube rooted and expressed as standardized score.
Executive function	Measured by TMT-A and TMT-B (available for 11 cases). Expressed as average of age-, sex-normalized z-scores.
BPF	Brain volume (available for 47 cases).
PSMD	Not available.
Blood NfL	Not available.
Remarks	-

**Cohort 8**	**Milan cohort 1**
References	**-**
Used as	Validation cohort
N	106
Setting	Patients and CADASIL-type-positive family members were recruited to the Istituto Neurologico Carlo Besta in Milan, and prospectively characterized
In- and exclusion criteria	Included were individuals with a *NOTCH3*^{cys} variant, or individuals with evidence of positive GOMs on skin biopsy, who were aged ≥ 18 years, and able to express informed consent. Excluded were patients who were not able to participate due to severe disability or who had contraindications for neuroimaging
Global cognition	Not available.
Executive function	Not available.
BPF	Not available.
PSMD	Not available.
Blood NfL	Not available.
Remarks	-

**Cohort 9**	**Sevilla cohort**
References	n/a
Used as	Validation cohort
N	36
Setting	Neurology department of regional hospital.
In- and exclusion criteria	Included were adult individuals with a *NOTCH3*^{cys} variant or with a well-documented cysteine-sparing *NOTCH3* variant, and MRI available, both symptomatic as well as presymptomatic family members diagnosed through family screening of the *NOTCH3* variant.

© 2024 Gravesteijn G et al. *JAMA Neurology.*

Global cognition	Not available.
Executive function	Not available.
BPF	Not available.
PSMD	Not available.
Blood NfL	Not available.
Remarks	-

**Cohort 10**	**Stockholm cohort**
References	-
Used as	Validation cohort
N	20
Setting	Both symptomatic CADASIL-patients and family members without clinical symptoms (*NOTCH3*^{cys}-mutation carriers) are recruited to an ongoing study called CADASIL in Sweden. The data for this article is from a subgroup. The participants have undergone extensive semistructured systematic annual interviews and their medical records including imaging results have been reviewed.
In- and exclusion criteria	Included were symptomatic CADASIL patients and asymptomatic individuals having a CADASIL-type NOTCH3 variant, who were aged ≥18 years and with informed consent.
Global cognition	Not available.
Executive function	Not available.
BPF	Not available.
PSMD	Not available.
Blood NfL	Not available.
Remarks	The Swedish cohort (Stockholm cohort) was recruited within the study CADASIL in Sweden, which is approved by Swedish Ethical Review Authority (Drn 2019-03851).

**Cohort 11**	**Osaka cohort**
References	doi.org/10.1002/ana.26916
Used as	Validation cohort
N	51
Setting	We retrospectively included consecutive patients with CADASIL admitted to the National Cerebral and Cardiovascular Center (NCVC) between January 2022 and January 2023.
In- and exclusion criteria	Included were patients with CADASIL who signed a comprehensive NCVC biobank consent form. Excluded were patients who withdrew the consent.
Global cognition	Measured by MOCA (available for all cases). For analyses, values were reflected (31-score) and then cube rooted and expressed as standardized score.
Executive function	Measured by Trail Making Test part A and B (available for all cases).
BPF	Not available.
PSMD	Not available.
Blood NfL	Not available.
Remarks	Supported by the Japan Agency for Medical Research and Development

**Cohort 12**	**Barcelona cohort**
References	doi.org/10.1038/s41598-021-86349-1
Used as	Validation cohort
N	21
Setting	"CADAGENIA" is a prospective registry of patients with mutations in NOTCH3, consecutively recorded since 2017 from different parts of Spain, mostly in Catalonia (Hospital de la Santa Creu i Sant Pau and Hospital Vall d'Hebron, Barcelona) Epidemiological data, blood analyses, cognition and neuroimaging profiles and skin biopsies are registered. We have a collection of samples registered in the Instituto Nacional de Salud Carlos III (C0005403).
In- and exclusion criteria	Included were patients over 18 years old with a *NOTCH3*^{cys} variant or with a well-documented cysteine-sparing *NOTCH3* variant. Excluded were individuals who were minors or who did not have a *NOTCH3*^{cys} variant.
Global cognition	Not available.

© 2024 Gravesteijn G et al. *JAMA Neurology.*

Executive function	Not available.
BPF	Not available.
PSMD	Not available.
Blood NfL	Not available.
Remarks	Supported by Asociacion CADASIL España

**Cohort 13**	**Newcastle cohort**
References	doi: 10.1111/bpa.12177
Used as	Validation cohort
N	22
Setting	Originally patients and pathology cases were referred to Newcastle MRC Neurochemical Pathology Unit and Centre for Brain Ageing and Vitality for *NOTCH3* analysis. Subsequently all positive cases were enrolled in clinicpathological longitudinal study in parallel the CogFAST (cognitive function after stroke) study.
In- and exclusion criteria	Included were individuals with a $NOTCH3^{cys}$ variant or with a well-documented cysteine-sparing *NOTCH3* variant, and relevant SVD and WMH features.
Exclusion criteria	-
Global cognition	Not available.
Executive function	Not available.
BPF	Not available.
PSMD	Not available.
Blood NfL	Not available.
Remarks	Newcastle cohort has several brain donoations which are retained in the Newcastle Brain Tissue Resource (NBTR). Supported by the Medical Research Council (UK).)

**Cohort 14**	**Glasgow cohort**
References	**-**
Used as	Validation cohort
N	97
Setting	Patients reviewed in the Neurovascular outpatient clinic (undergoing MRI between 2004 and 2019 as part of routine care)
In- and exclusion criteria	Included were adult individuals with a *NOTCH3* pathogenic variant. Individuals for whom no MRI data was available were excluded.
Global cognition	Not available.
Executive function	Not available.
BPF	Not available.
PSMD	Not available.
Blood NfL	Not available.
Remarks	-

**Cohort 15**	**Paris cohort**
References	https://doi.org/10.1177/0271678X221126280
Used as	Validation cohort
N	435
Setting	Patients and *NOTCH3*cys-positive family members were recruited between 2003 and 2020 at the French National Referral Center for rare cerebrovascular diseases in France (www.cervco.fr)
In- and exclusion criteria	Included were individuals with a $NOTCH3^{cys}$ variant with age ≥ 18 years. Excluded were individuals who were unable to provide informed consent.
Global cognition	Not available.
Executive function	Not available.
BPF	Not available.
PSMD	Not available.
Blood NfL	Not available.
Remarks	

**Cohort 16**	**Milan cohort 2**
References	**-**
Used as	Validation cohort
N	8
Setting	Outpatient neurology vascular cognitive decline (VAS-COG) clinic (years 2018-2024)
In- and exclusion criteria	Included were individuals with a *NOTCH3*^{cys} variant or with a well-documented cysteine-sparing *NOTCH3* variant, aged ≥ 18 years, regardless of their clinical symptoms or severity of leukoencephalopathy, who were willing to participate to clinical and neuroimaging assessment. Excluded were individuals who were not able to participate to the clinical and/or neuroimaging assessment due to severe disability; any other contraindication for neuroimaging.
Global cognition	Not available.
Executive function	Not available.
BPF	Not available.
PSMD	Not available.
Blood NfL	Not available.
Remarks	-

**Cohort 17**	**Siena cohort**
References	**-**
Used as	Validation cohort
N	20
Setting	Not available.
In- and exclusion criteria	Not available.
Global cognition	Measured by MMSE (available for 13 cases). For analyses, values were reflected (31-score) and then cube rooted and expressed as standardized score.
Executive function	Not available.
BPF	Not available.
PSMD	Not available.
Blood NfL	Not available.
Remarks	

**Cohort 18**	**Coimbra cohort**
References	doi.org/10.1016/j.cccb.2023.100186
Used as	Validation cohort
N	15
Setting	Neurology service within the hospital and university center of Coimbra. The patients were consecutively selected from a cohort of CADASIL patients regularly followed in the cerebrovascular risk consultation.
In- and exclusion criteria	Included were individuals who fulfilled the following criteria: (i) a previous clinical diagnosis of CADASIL, confirmed by genetic testing, and (ii) age below 66 years old; (iii) a cerebral MRI routinely performed in the previous 12 months with quantification of WMH load. Excluded were individuals with one of the following: (i) presence of dementia (ii) significant changes in visual or auditory acuity that could compromise the performance of the assessment; (iii) unstable clinical status (e.g. delirium, etc.).
Global cognition	Measured by MOCA (available in all cases). For analyses, values were reflected (31-score) and then cube rooted and expressed as standardized score.
Executive function	Measures by DS (available in all cases). DS samples were transformed
BPF	Not available.
PSMD	Not available.
Blood NfL	Not available.
Remarks	-

**Cohort 19**	**Melbourne cohort**
References	-
Used as	Validation cohort
N	6

© 2024 Gravesteijn G et al. *JAMA Neurology.*

Setting	Not available.
In- and exclusion criteria	Not available.
Global cognition	Not available.
Executive function	Not available.
BPF	Not available.
PSMD	Not available.
Blood NfL	Not available.
Remarks	

**Cohort 20**	**Florence cohort**
References	Doi.org/10.3233/JAD-141569
Used as	Validation cohort
N	89
Setting	VAS-COG out-patient clinic dedicated to patients affected by sporadic and inherited cerebral small vessel disease.
In- and exclusion criteria	Included were CADASIL patients and family members with a *NOTCH3*^{cys} variant, aged ≥18 years. Excluded were individuals who were unable to complete neuroimaging.
Global cognition	Measured by MMSE (available in 27 cases) and MOCA (available in 50 cases). For analyses, values were reflected (31-score) and then cube rooted and expressed as standardized score.
Executive function	Measures by SDMT (available for 60 cases). Raw SDMT values were normalized to z-scores.
BPF	Not available.
PSMD	Not available.
Blood NfL	Not available.
Remarks	

**Cohort 21**	**Jacksonville cohort**
References	-
Used as	Validation cohort
N	62
Setting	Cases identified by search of the integrated electronic medical records system (EPIC) used across the Mayo Clinic.
In- and exclusion criteria	Included were individuals with a *NOTCH3*^{cys} variant.
Global cognition	Measured by MOCA (available in all cases). For analyses, values were reflected (31-score) and then cube rooted and expressed as standardized score.
Executive function	Not available in sufficient cases.
BPF	Not available.
PSMD	Not available.
Blood NfL	Not available.
Remarks	-

**Cohort 22**	**Brasilia cohort**
References	doi.org/10.1055/s-0042-1758756
Used as	Validation cohort
N	19
Setting	Case series from 6 rehabilitation centers in Brazil, between 2002 and 2019.
In- and exclusion criteria	Included were individuals with CADASIL and molecular testing showing a *NOTCH3*^{cys} variant or with a well-documented cysteine-sparing *NOTCH3* variant. Relatives with a *NOTCH3* variant were also included, but not actively recruited.
Global cognition	MOCA available, given the small sample size not shown in Supplementary data 6B.
Executive function	Not available.
BPF	Not available.

© 2024 Gravesteijn G et al. *JAMA Neurology.*

PSMD	Not available.
Blood NfL	Not available.
Remarks	-

**Cohort 23**	**Beijing cohort**
References	doi.org/10.1002/acn3.52214
Used as	Validation cohort
N	105
Setting	Individuals were recruited at the neurology department of the Peking university first hospital.
In- and exclusion criteria	Included were individuals with a confirmed genetic or histopathological diagnosis of CADASIL and age > 18 years.
Global cognition	Measured by MMSE (available in all cases). For analyses, values were reflected (31-score) and then cube rooted and expressed as standardized score.
Executive function	Measured by TMT-A and TMT-B (available for 95 cases). Expressed as average of age-, sex-normalized z-scores.
BPF	Not available.
PSMD	Not available.
Blood NfL	Not available.
Remarks	-

**Cohort 24**	**UK Biobank cohort**
References	doi.org/10.1038/s41586-018-0579-z
Used as	Population-based validation cohort
N	101
Setting	Community-dwelling individuals who are considered healthy, recruited to the UK Biobank.
In- and exclusion criteria	Included were individuals in the UK Biobank with a $NOTCH3^{cys}$ variant with neuroimaging available.
Global cognition	Not available.
Executive function	The Trail Making Test obtained as parts of the UK Biobank is not similar to the traditional Trail Making Test, unabling us to obtain age, sex and educational level standardized z-scores. Therefore, z-scores were obtained by standardizing the data to itself.
BPF	Available.
PSMD	Available.
Blood NfL	Not available.
Remarks	Ischemic stroke and intracerebral hemorrhage data was obtained by assessing the ICD10 codes for I63 (ischemic stroke) and I61 (intracerebral hemorrhage). This research has been conducted using the UK Biobank Resource under Application Number 74162.

© 2024 Gravesteijn G et al. *JAMA Neurology.*

eTable 1: Selection of Features for the NOTCH3-SVD Staging System

Candidate features	Criterion *i*: readily and uniformly obtained	Criterion *ii:* present in 1/3 of patients	Criterion *iii:* risk increases with age	Fulfilling all criteria
Clinical features				
Migraine	●	●	✕	
Migraine with aura	●	●	✕	
TIA	✕			
Stroke	✕			
'Being symptomatic'	✕			
Apathy	✕			
Depression	●	●	✕	
Disability (mRS)	●	●	●	●
Gait disturbances	●	●	●	●
Bedridden	●	●	●	●
Seizures/epilepsy	●	✕		
Cognitive impairment	✕			
Dementia	✕			
Encephalopathy	●	✕		
Death	●	●	●	●
Neuroimaging features				
Lacunes, count	●	●	●	●
Microbleed, count	●	●	●	●
Fazekas dwm, score	●	●	●	●
ICH	●	✕		
Brain atrophy (%BPF)	✕			
Lacune (volume)	✕			
WMH (volume)	✕			
PSMD	✕			
ePVS, count/score	✕			

Dots indicate that a certain feature fulfills the criterion, while a cross indicated that the criterion is not fulfilled.

© 2024 Gravesteijn G et al. *JAMA Neurology.*

eTable 2: Microbleeds Do Not Improve NOTCH3-SVD Staging System

(A)	Model 1: NOTCH3-SVD staging system	Model 2: Cerebral microbleed (CMB) presence	Model 3: NOTCH3-SVD staging system + CMB presence	Delta BIC Model 3 minus Model 1
**Bayesian information criterion (BIC)**				
Unadjusted				
PSMD	343	415	342	-2
BPF	295	328	300	5
NfL	130	125	131	0
Global Cognition	371	382	373	2
Processing speed	357	399	362	5
Stroke (dichotomous)	176	214	195	19
Adjusted for age and sex				
PSMD	317	362	319	3
BPF	239	247	242	3
NfL	134	122	136	2
Global Cognition	370	374	374	4
Processing speed	366	407	372	5
Stroke (dichotomous)	200	232	205	4
(B)	Model 1: NOTCH3-SVD staging system	Model 2: Cerebral microbleed (CMB) presence	Model 3: NOTCH3-SVD staging system + CMB presence	*P* Model 1 vs. Model 3
**Adjusted R^2 values**				
Unadjusted				
PSMD	0.57	0.27	0.58	0.05
BPF	0.33	0.11	0.32	0.99
NfL	0.27	0.15	0.30	0.36
Global Cognition	0.25	0.11	0.26	0.47
Processing speed	0.31	0.04	0.30	0.99
Adjusted for age and sex				
PSMD	0.65	0.49	0.65	0.58
BPF	0.51	0.44	0.52	0.87
NfL	0.30	0.27	0.31	0.70
Global Cognition	0.29	0.19	0.29	0.99
Processing speed	0.30	0.04	0.30	0.99

(A) The Bayesian Information Criterion (BIC) is shown for three models comparing the performance of the NOTCH3-SVD staging system (model 1), the a model with the presence of cerebral microbleeds (CMB)(dichotomous, model 2) and a combination of the NOTCH3-SVD staging system and CMB presence (model 3) , with and without correcting for age and sex. BIC takes the number of terms in the statical model into account, allowing for comparison of the 2-level CMB presence model with the 9-level NOTCH3-SVD staging system. The lowest BIC values represent the best model fit (underlined BIC values). The combined model of NOTCH3-SVD staging system with CMB presence (model 3) did not have lower BIC values and did thus not perform better than NOTCH3-SVD staging system alone (model 1). (B) Adjusted R^2 values of model 1, model 2 and model 3 are shown with and without correcting for age and sex. Higher R^2 values represent a better model fit. Adding CMB presence to the NOTCH3-SVD staging system did improve the statistical model (F-test, $P_{\text{Model 3 vs. Model 1}}$). *P*-values were Bonferoni corrected for multiple testing.

© 2024 Gravesteijn G et al. *JAMA Neurology.*

eTable 3: Cohort Characteristics

eTable 3a – Cohort Characteristics

	Leiden DiViNAS	Leiden FU18	Cambridge	Munich	Taiwan	Jeju	Seoul	Milan
N	195	41	149	53	132	87	135	108
Age, mean (sd)	52.4 (12.2)	45.8 (10.4)	49.4 (11.6)	53.7 (10.6)	61.1 (13.0)	62.5 (12.7)	54.4 (11.4)	53.3 (13.2)
Female, n (%)	102 (52%)	22 (54%)	86 (58%)	36 (68%)	75 (57%)	-	77 (57%)	56 (52%)
NOTCH3 risk category, n (%)								
HR	112 (57%)	37 (90%)	120 (81%)	33 (87%)	8 (6%)	2 (2%)	81 (60%)	66 (61%)
MR	78 (40%)	4 (10%)	25 (17%)	5 (13%)	122 (94%)	85 (98%)	52 (39%)	33 (31%)
LR	5 (3%)	0 (0%)	4 (3%)	0 (0%)	0 (0%)	0 (0%)	2 (1%)	9 (8%)
UR	0 (0%)	0 (0%)	0 (0%)	0 (0%)	0 (0%)	0 (0%)	0 (0%)	0 (0%)
na	0 (0%)	0 (0%)	0 (0%)	0 (0%)	0 (0%)	0 (0%)	0 (0%)	0 (0%)
Fazekas DWM score, n (%)								
score 0	9 (5%)	3 (7%)	3 (2%)	0 (0%)	14 (11%)	4 (5%)	3 (2%)	5 (5%)
score 1	29 (15%)	3 (7%)	18 (12%)	3 (6%)	21 (16%)	9 (10%)	14 (10%)	10 (9%)
score 2-3	157 (81%)	35 (85%)	128 (86%)	50 (94%)	97 (73%)	74 (85%)	118 (87%)	93 (86%)
Lacune count, n (%)								
0	87 (45%)	12 (29%)	42 (28%)	16 (30%)	33 (25%)	18 (21%)	34 (25%)	36 (33%)
1-4	46 (24%)	11 (27%)	73 (49%)	9 (17%)	14 (11%)	32 (37%)	54 (40%)	35 (32%)
>5	62 (32%)	18 (44%)	34 (23%)	28 (53%)	85 (64%)	37 (43%)	47 (35%)	37 (34%)
modified Rankin Scale, n (%)								
0-2	169 (87%)	30 (73%)	137 (92%)	48 (91%)	77 (58%)	73 (84%)	125 (93%)	78 (72%)
3	21 (11%)	6 (15%)	11 (7%)	4 (8%)	26 (20%)	4 (5%)	8 (6%)	14 (13%)
4	5 (3%)	4 (10%)	1 (1%)	1 (2%)	23 (17%)	6 (7%)	2 (1%)	15 (14%)
5	0 (0%)	1 (2%)	0 (0%)	0 (0%)	6 (5%)	4 (5%)	0 (0%)	1 (1%)
History of stroke, n (%)	55 (28%)	23 (56%)	60 (40%)	24 (45%)	70 (53%)	38 (44%)	68 (50%)	37 (34%)
History of ICH, n (%)	1 (1%)	0 (0%)	3 (2%)	0 (0%)	11 (8%)	15 (17%)	2 (1%)	7 (6%)
NOTCH3-SVD stage, n (%)								
stage 0	9 (5%)	3 (7%)	3 (2%)	0 (0%)	14 (11%)	4 (5%)	2 (1%)	5 (5%)
stage 1A	26 (13%)	3 (7%)	12 (8%)	3 (6%)	15 (11%)	7 (8%)	8 (6%)	9 (8%)
stage 1B	48 (25%)	6 (15%)	27 (18%)	13 (25%)	4 (3%)	7 (8%)	22 (16%)	21 (19%)
stage 2A	44 (23%)	10 (24%)	71 (48%)	8 (15%)	12 (9%)	27 (31%)	52 (39%)	28 (26%)
stage 2B	42 (22%)	8 (20%)	24 (16%)	24 (45%)	32 (24%)	28 (32%)	41 (30%)	15 (14%)
stage 3A	21 (11%)	6 (15%)	11 (7%)	4 (8%)	26 (20%)	4 (5%)	8 (6%)	14 (13%)
stage 3B	5 (3%)	4 (10%)	1 (1%)	1 (2%)	23 (17%)	6 (7%)	2 (1%)	15 (14%)
stage 4A	0 (0%)	1 (2%)	0 (0%)	0 (0%)	6 (5%)	4 (5%)	0 (0%)	1 (1%)
Global cognition, median (IQR)	25 (5)	28 (3)	25 (5)	30 (1)	29 (6)	22 (12)	26 (8)	-
Processing speed, mean (sd)	-0.2 (1.4)	-1.6 (2.1)	-	-1.6 (3)	-2 (2.3)	-	-2 (4.7)	-
BPF, mean (sd)	70.1 (3.9)	82.2 (3)	76.3 (6.3)	78.2 (4.6)	83.3 (5.3)	76.3 (4.9)	-	-
PSMD, median (IQR)	0.34 (0.24)	-	-	0.54 (0.28)	0.44 (0.44)	-	-	-
Serum NfL pg/ml, median (IQR)	10.1 (10.3)	5.7 (9.1)	-	41.7 (46.9)	-	-	-	-

© 2024 Gravesteijn G et al. *JAMA Neurology.*

eTable 3b – Cohort Characteristics (continued)

	Sevilla	Stockholm	Osaka	Barcelona	Newcastle	Glasgow	Paris	Milan2
N	36	20	51	23	22	97	435	8
Age, mean (sd)	52.8 (13.1)	55.6 (14.2)	54.0 (8.6)	49.6 (12.2)	48.3 (7.6)	47.5 (11.5)	51.9 (12.0)	50.6 (6.3)
Female, n (%)	24 (67%)	-	21 (41%)	11 (48%)	5 (23%)	54 (56%)	-	6 (75%)
NOTCH3 risk category, n (%)								
HR	21 (58%)	16 (80%)	43 (84%)	18 (78%)	20 (91%)	82 (85%)	-	5 (63%)
MR	14 (39%)	4 (20%)	7 (14%)	4 (17%)	1 (5%)	5 (5%)	-	2 (25%)
LR	1 (3%)	0 (0%)	1 (2%)	1 (4%)	1 (5%)	9 (9%)	-	1 (13%)
UR	0 (0%)	0 (0%)	0 (0%)	0 (0%)	0 (0%)	0 (0%)	-	0 (0%)
na	0 (0%)	0 (0%)	0 (0%)	0 (0%)	0 (0%)	0 (0%)	-	0 (0%)
Fazekas DWM score, n (%)								
score 0	3 (8%)	0 (0%)	0 (0%)	1 (4%)	0 (0%)	4 (4%)	-	0 (0%)
score 1	13 (36%)	2 (10%)	0 (0%)	3 (13%)	3 (14%)	14 (14%)	-	2 (25%)
score 2-3	20 (56%)	18 (90%)	51 (100%)	19 (83%)	19 (86%)	79 (81%)	-	6 (75%)
Lacune count, n (%)								
0	20 (56%)	5 (25%)	5 (10%)	10 (43%)	3 (14%)	39 (40%)	-	3 (38%)
1-4	8 (22%)	4 (20%)	21 (41%)	3 (13%)	12 (55%)	27 (28%)	-	3 (38%)
>5	8 (22%)	11 (55%)	25 (49%)	10 (43%)	7 (32%)	31 (32%)	-	2 (25%)
modified Rankin Scale, n (%)								
0-2	34 (94%)	16 (80%)	48 (94%)	21 (91%)	0 (0%)	90 (93%)	369 (85%)	8 (100%)
3	1 (3%)	2 (10%)	3 (6%)	1 (4%)	0 (0%)	4 (4%)	39 (9%)	0 (0%)
4	1 (3%)	2 (10%)	0 (0%)	1 (4%)	0 (0%)	3 (3%)	14 (3%)	0 (0%)
5	0 (0%)	0 (0%)	0 (0%)	0 (0%)	22 (100%)	0 (0%)	13 (3%)	0 (0%)
History of stroke, n (%)	19 (53%)	-	30 (59%)	5 (22%)	16 (73%)	43 (48%)	-	3 (38%)
History of ICH, n (%)	0 (0%)	-	4 (8%)	0 (0%)	1 (5%)	3 (3%)	-	0 (0%)
NOTCH3-SVD stage, n(%)								
stage 0	3 (8%)	0 (0%)	0 (0%)	1 (4%)	0 (0%)	4 (4%)	4 (1%)	0 (0%)
stage 1A	10 (28%)	1 (5%)	0 (0%)	2 (9%)	2 (9%)	11 (11%)	15 (3%)	2 (25%)
stage 1B	7 (19%)	4 (20%)	4 (8%)	7 (30%)	1 (5%)	24 (25%)	71 (16%)	1 (13%)
stage 2A	8 (22%)	4 (20%)	21 (41%)	3 (13%)	12 (55%)	24 (25%)	105 (24%)	3 (38%)
stage 2B	6 (17%)	7 (35%)	23 (45%)	8 (35%)	5 (23%)	27 (28%)	174 (40%)	2 (25%)
stage 3A	1 (3%)	2 (10%)	3 (6%)	1 (4%)	2 (9%)	4 (4%)	39 (9%)	0 (0%)
stage 3B	1 (3%)	2 (10%)	0 (0%)	1 (4%)	0 (0%)	3 (3%)	14 (3%)	0 (0%)
stage 4A	0 (0%)	0 (0%)	0 (0%)	0 (0%)	0 (0%)	0 (0%)	13 (3%)	0 (0%)
Global cognition, median (IQR)	-	-	25 (5)	-	-	-	-	-
Processing speed, mean (sd)	-	-	-0.9 (1.6)	-	-	-	-	-
BPF, mean (sd)	-	-	-	-	-	-	-	-
PSMD, median (IQR)	-	-	-	-	-	-	-	-
Serum NfL pg/ml, median (IQR)	-	-	-	-	-	-	-	-

eTable 3c – Cohort Characteristics (continued)

	Siena	Coimbra	Melbourne	Florence	Jacksonville	Brasilia	Beijing
N	20	15	6	89	62	19	105
Age, mean (sd)	46.5 (11.9)	-	-	60.4 (15.9)	57.8 (12.9)	-	45.0 (10.2)
Female, n (%)	8 (40%)	-	-	58 (65%)	42 (68%)	12 (63%)	47 (45%)
NOTCH3 risk category, n (%)							
HR	9 (45%)	-	-	30 (34%)	36 (59%)	18 (95%)	72 (69%)
MR	8 (40%)	-	-	33 (38%)	22 (36%)	1 (5%)	31 (30%)
LR	3 (15%)	-	-	24 (28%)	3 (5%)	0 (0%)	1 (1%)
UR	0 (0%)	-	-	0 (0%)	0 (0%)	0 (0%)	1 (1%)
na	0 (0%)	-	-	0 (0%)	0 (0%)	0 (0%)	0 (0%)
Fazekas DWM score, n (%)							
score 0	4 (20%)	1 (7%)	0 (0%)	6 (7%)	0 (0%)	1 (5%)	5 (5%)
score 1	4 (20%)	1 (7%)	0 (0%)	18 (20%)	9 (15%)	4 (21%)	31 (30%)
score 2-3	12 (60%)	13 (87%)	6 (100%)	65 (73%)	53 (85%)	14 (74%)	69 (66%)
Lacune count, n (%)							
0	12 (60%)	9 (60%)	1 (17%)	30 (34%)	25 (40%)	6 (32%)	29 (28%)
1-4	4 (20%)	1 (7%)	3 (50%)	29 (33%)	27 (44%)	4 (21%)	14 (13%)
>5	4 (20%)	5 (33%)	2 (33%)	30 (34%)	10 (16%)	9 (47%)	62 (59%)
modified Rankin Scale, n (%)							
0-2	17 (85%)	13 (87%)	6 (100%)	68 (76%)	53 (85%)	14 (74%)	92 (88%)
3	1 (5%)	1 (7%)	0 (0%)	9 (10%)	6 (10%)	3 (16%)	11 (10%)
4	2 (10%)	0 (0%)	0 (0%)	12 (13%)	3 (5%)	0 (0%)	2 (2%)
5	0 (0%)	1 (7%)	0 (0%)	0 (0%)	0 (0%)	2 (11%)	0 (0%)
History of stroke, n (%)	9 (45%)	6 (40%)	-	29 (33%)	47 (76%)	15 (79%)	70 (67%)
History of ICH, n (%)	2 (10%)	2 (13%)	-	7 (8%)	2 (3%)	1 (5%)	15 (14%)
NOTCH3-SVD stage, n (%)							
stage 0	3 (15%)	1 (7%)	0 (0%)	6 (7%)	0 (0%)	1 (5%)	5 (5%)
stage 1A	1 (5%)	1 (7%)	0 (0%)	13 (15%)	5 (8%)	3 (16%)	19 (18%)
stage 1B	6 (30%)	7 (47%)	0 (0%)	11 (12%)	20 (32%)	2 (11%)	5 (5%)
stage 2A	3 (15%)	1 (7%)	0 (0%)	23 (26%)	22 (35%)	4 (21%)	14 (13%)
stage 2B	4 (20%)	3 (20%)	1 (17%)	15 (17%)	6 (10%)	4 (21%)	49 (47%)
stage 3A	1 (5%)	1 (7%)	3 (50%)	9 (10%)	6 (10%)	3 (16%)	11 (10%)
stage 3B	2 (10%)	0 (0%)	2 (33%)	12 (13%)	3 (5%)	0 (0%)	2 (2%)
stage 4A	0 (0%)	1 (7%)	0 (0%)	0 (0%)	0 (0%)	2 (11%)	0 (0%)
Global cognition, median (IQR)	30 (a2)	23 (8)	-	26 (6)	25 (6)	-	29 (4)
Processing speed, mean (sd)	-	-2.1 (2)	-	-0.6 (0.9)	-	-	-2.7 (1.8)
BPF, mean (sd)	-	-	-	-	-	-	-
PSMD, median (IQR)	-	-	-	-	-	-	-
Serum NfL pg/ml, median (IQR)	-	-	-	-	-	-	-

© 2024 Gravesteijn G et al. *JAMA Neurology.*

eTable 4: Effect Sizes for the Association Between the NOTCH3-SVD Stages and Age, Global Cognition, Processing Speed, BPF, PSMD, and NfL

		Age	P_{age}	Sex	P_{sex}	NOTCH3-SVD stage								
						0	1A	1B	2A	2B	3A	3B	4A	*P*-value
<u>**DISCOVERY COHORT**</u>														
**Global cognition**														
Unadj.	St. β					0.000	0.028	-0.153	-0.333	-0.468	-1.118	-1.682		**<.001**
	s.e.					0.000	0.236	0.222	0.226	0.226	0.249	0.367		
Adj.	St. β	0.014	0.001	0.089	0.348	0.000	0.094	0.036	-0.064	-0.136	-0.768	-1.291		**<.001**
	s.e.	0.004		0.094		0.000	0.232	0.228	0.237	0.242	0.265	0.376		
**Processing speed**														
Unadj.	St. β					0.000	-0.225	-0.116	-0.099	-0.343	-1.084	-1.781		**<.001**
	s.e.					0.000	0.214	0.201	0.202	0.203	0.220	0.308		
Adj.	St. β	-0.001	0.781	0.077	0.914	0.000	-0.200	-0.068	-0.057	-0.317	-1.052	-1.759		**<.001**
	s.e.	0.004		0.084		0.000	0.216	0.212	0.218	0.223	0.242	0.330		
**BPF**														
Unadj.	St. β					0.000	-0.153	-0.236	-0.412	-0.697	-1.076	-1.457		**<.001**
	s.e.					0.000	0.182	0.171	0.172	0.173	0.188	0.263		
Adj.	St. β	0.008	0.592	0.004	0.985	0.000	-0.101	-0.034	-0.068	-0.204	-0.557	-0.809		**<.001**
	s.e.	0.016		0.224		0.000	0.156	0.153	0.157	0.161	0.175	0.238		
**PSMD**														
Unadj.	St. β					0.000	-0.298	-0.980	-1.308	-1.883	-2.038	-2.985		**<.001**
	s.e.					0.000	0.223	0.211	0.214	0.214	0.235	0.347		
Adj.	St. β	0.020	0.156	0.220	0.272	0.000	-0.146	-0.584	-0.808	-1.340	-1.442	-2.345		**<.001**
	s.e.	0.014		0.199		0.000	0.204	0.202	0.210	0.213	0.234	0.331		
**Serum NfL**														
Unadj.	St. β					0.000	0.150	-0.087	-0.513	-0.793	-0.413	-1.559		**<.001**
	s.e.					0.000	0.573	0.558	0.557	0.553	0.624	0.764		
Adj.	St. β	0.014	0.056	-0.016	0.914	0.000	0.187	0.106	-0.216	-0.465	-0.105	-1.034		.74
	s.e.	0.007		0.143		0.000	0.575	0.563	0.575	0.571	0.636	0.799		

In the discovery cohort, the associations between the NOTCH3-SVD staging system (as categorical independent variable) and BPF, PSMD, global cognition, processing speed, and serum NfL were dertermined using linear regression analyses with and without adjusting for age and sex. Per outcome measure, the unadjusted standardized beta's (Unadj. St. β) and its standard errors (s.e.) for the stages of the NOTCH3-SVD staging system (stage 0 being the reference category), as well as the adjusted standardize beta's (Unadj. St. β) and its standards errors (s.e.) for the NOTCH3-SVD staging system, age and sex.

© 2024 Gravesteijn G et al. *JAMA Neurology.*

		Age	P_{age}	Sex	P_{sex}	NOTCH3-SVD stage 0	1A	1B	2A	2B	3A	3B	4A	*P*-value
**VALIDATION COHORTS**														
**Global cognition**														
Unadj.	St. β					0.000	-0.098	-0.323	-0.404	-0.651	-1.336	-2.189	-2.223	**<.001**
	s.e.					0.000	0.169	0.165	0.153	0.150	0.171	0.191	0.373	
Adj.	St. β	0.008	0.011	0.000	0.999	0.000	0.003	-0.095	-0.146	-0.358	-0.994	-1.919	-1.493	**<.001**
	s.e.	0.003		0.065		0.000	0.205	0.211	0.209	0.216	0.240	0.264	0.741	
**Processing speed**														
Unadj.	St. β					0.000	-0.158	-0.374	-0.365	-0.867	-1.636	-2.180	-1.905	**<.001**
	s.e.					0.000	0.180	0.181	0.168	0.164	0.185	0.213	0.581	
Adj.	St. β	-0.001	0.752	0.064	0.367	0.000	-0.118	-0.266	-0.296	-0.794	-1.568	-2.118	-2.425	**<.001**
	s.e.	0.004		0.071		0.000	0.186	0.195	0.189	0.191	0.218	0.243	0.808	
**BPF**														
Unadj.	St. β					0.000	-0.181	-0.231	-0.496	-0.695	-1.135	-1.241	-1.882	**<.001**
	s.e.					0.000	0.193	0.188	0.173	0.171	0.193	0.210	0.357	
Adj.	St. β	-0.015	0.004	-0.236	0.004	0.000	-0.114	-0.004	-0.127	-0.170	-0.685	-0.724	-0.528	**.005**
	s.e.	0.004		0.081		0.000	0.205	0.211	0.209	0.216	0.240	0.264	0.741	
**PSMD**														
Unadj.	St. β					0.000	-0.322	-1.354	-1.400	-1.998	-2.791	-2.987		**<.001**
	s.e.					0.000	0.175	0.187	0.174	0.150	0.181	0.189		
Adj.	St. β	0.016	0.000	0.129	0.125	0.000	-0.139	-1.007	-0.937	-1.533	-2.298	-2.514		**<.001**
	s.e.	0.004		0.083		0.000	0.173	0.198	0.202	0.183	0.211	0.218		
**Serum NfL**														
Unadj.	St. β					0.000	-0.339	-0.653	-0.863	-1.237	-1.761	-2.485	-2.572	**<.001**
	s.e.					0.000	0.439	0.393	0.390	0.385	0.408	0.449	0.708	
Adj.	St. β	0.032	0.000	0.065	0.619	0.000	-0.099	0.035	-0.025	-0.308	-0.677	-1.431	-1.601	**.002**
	s.e.	0.008		0.131		0.000	0.411	0.406	0.419	0.427	0.467	0.494	0.705	

In the validation cohorts, the associations between the NOTCH3-SVD staging system (as categorical independent variable) and BPF, PSMD, global cognition, processing speed, and serum NfL were dertermined using linear mixed model analyses with cohort as random effect with and without adjusting for age and sex.

© 2024 Gravesteijn G et al. *JAMA Neurology*.

		Age	P_{age}	Sex	P_{sex}	NOTCH3-SVD stage 0	1A	1B	2A	2B	3A	3B	4A	*P*-value
**UK BIOBANK**														
**Processing speed**														
Unadj.	St. β					0.000	-0.424	0.441	0.007	-0.350				.11
	s.e.					0.000	0.288	0.354	0.387	0.882				
Adj.	St. β	0.057	<0.001	0.038	0.849	0.000	-0.129	0.321	0.079	-0.296				.63
	s.e.	0.016		0.199		0.000	0.277	0.326	0.355	0.815				
**BPF**														
Unadj.	St. β					0.000	0.297	0.029	-0.155	-1.798				.52
	s.e.					0.000	0.273	0.329	0.378	1.014				
Adj.	St. β	0.008	0.592	0.004	0.985	0.000	0.235	-0.125	-0.251	-1.905				.37
	s.e.	0.016		0.224		0.000	0.308	0.367	0.405	1.059				
**PSMD**														
Unadj.	St. β					0.000	-0.158	0.875	1.012	1.622				**<.001**
	s.e.					0.000	0.250	0.303	0.352	0.909				
Adj.	St. β	0.020	0.156	0.220	0.272	0.000	-0.005	1.062	1.121	1.614				**<.001**
	s.e.	0.014		0.199		0.000	0.275	0.324	0.367	0.923				

In the UK Biobank validation cohort, the associations between the NOTCH3-SVD staging system (as categorical independent variable) and BPF, PSMD, and processing speed were dertermined using linear analyses with and without adjusting for age and sex.

© 2024 Gravesteijn G et al. *JAMA Neurology*.

eTable 5: Statistical Comparison of the NOTCH3-SVD Staging System With the Previously Reported Anisetti System

(A)	Model 1: NOTCH3-SVD staging system	Model 2: Anisetti system	Model 3: NOTCH3-SVD staging system + Anisetti system	Delta BIC Model 1 vs. Model 2	
**Bayesian information criterion (BIC)**					
Unadjusted					
PSMD	343	420	355	-77	
BPF	295	323	306	-29	
NfL	130	129	140	1	
Global Cognition	371	399	384	-28	
Processing speed	357	395	365	-38	
Stroke (dichotomous)	176	185	189	-10	
Adjusted for age and sex					
PSMD	317	369	330	-52	
BPF	239	252	254	-13	
NfL	134	131	146	4	
Global Cognition	370	388	384	-18	
Processing speed	366	405	375	-39	
Stroke (dichotomous)	200	215	198	-15	
**(B)**	**Model 1: NOTCH3-SVD staging system**	**Model 2: Anisetti system**	**Model 3: NOTCH3-SVD staging system + Anisetti system**	***P* Model 3 vs. Model 2**	***P* Model 3 vs. Model 1**
**Adjusted R^2 values**					
Unadjusted					
PSMD	0.57	0.29	0.57	**<.001**	.99
BPF	0.33	0.17	0.33	**<.001**	.99
NfL	0.27	0.18	0.27	.22	.99
Global Cognition	0.25	0.06	0.25	**<.001**	.99
Processing speed	0.31	0.10	0.32	**<.001**	.37
Adjusted for age and sex					
PSMD	0.65	0.49	0.64	**<.001**	.99
BPF	0.51	0.45	0.51	**<.001**	.99
NfL	0.30	0.24	0.27	.99	.99
Global Cognition	0.29	0.16	0.28	**<.001**	.99
Processing speed	0.30	0.09	0.32	**<.001**	.37

(A) The Bayesian Information Criterion (BIC) is shown for three models comparing the performance of the NOTCH3-SVD staging system (model 1), the Anisetti system (model 2) and a combination of the NOTCH3-SVD staging system and the Anisetti model (model 3) , with and without correcting for age and sex. BIC takes the number of terms in the statical model into account, allowing for comparison of the 5-level Anisetti model with the 9-level NOTCH3-SVD staging system. Lower BIC values represent a better model fit (underlined BIC values). NOTCH3-SVD staging system, performed better than the Anisetti model and the combined model, both before and after correcting for age. The Anisetti model performed slightly better after correcting for age and sex in predicting NfL values. (B) Adjusted R^2 values of model 1, model 2 and model 3 are shown with and without correcting for age and sex. Higher R^2 values represent a better model fit. Adding the NOTCH3-SVD staging system to the Anisetti system did improve the statistical model (F-test, $P_{\text{Model3 vs. Model 2}}$), while adding the Anisetti system to the NOTCH3-SVD staging system did not improve the statistical model (F-test, $P_{\text{Model3 vs. Model1}}$). *P*-values were Bonferoni corrected for multiple testing.

© 2024 Gravesteijn G et al. *JAMA Neurology.*

eFigure 1: CADASIL and NOTCH3-SVD Features by Age

Potential disease hallmarks were tested against three criteria: (*i*) can be readily and uniformly obtained, (*ii*) are present in one-third of patients in the course of the disease, and (*iii*) the risk of developing the featureincreases with age. (A) Disease hallmarks that did not show considerable increase in prevalence with increasing age. These hallmarks were excluded from the staging staging system. (B,C) Clinical hallmarks and neuroimaging hallmarks which showed an increasing prevalence with age. While a history of TIA, stroke and vascular cognitive impairment were progressive hallmarks and were present in the majority of disease courses, they were excluded as they did not fulfill criterion *i*.

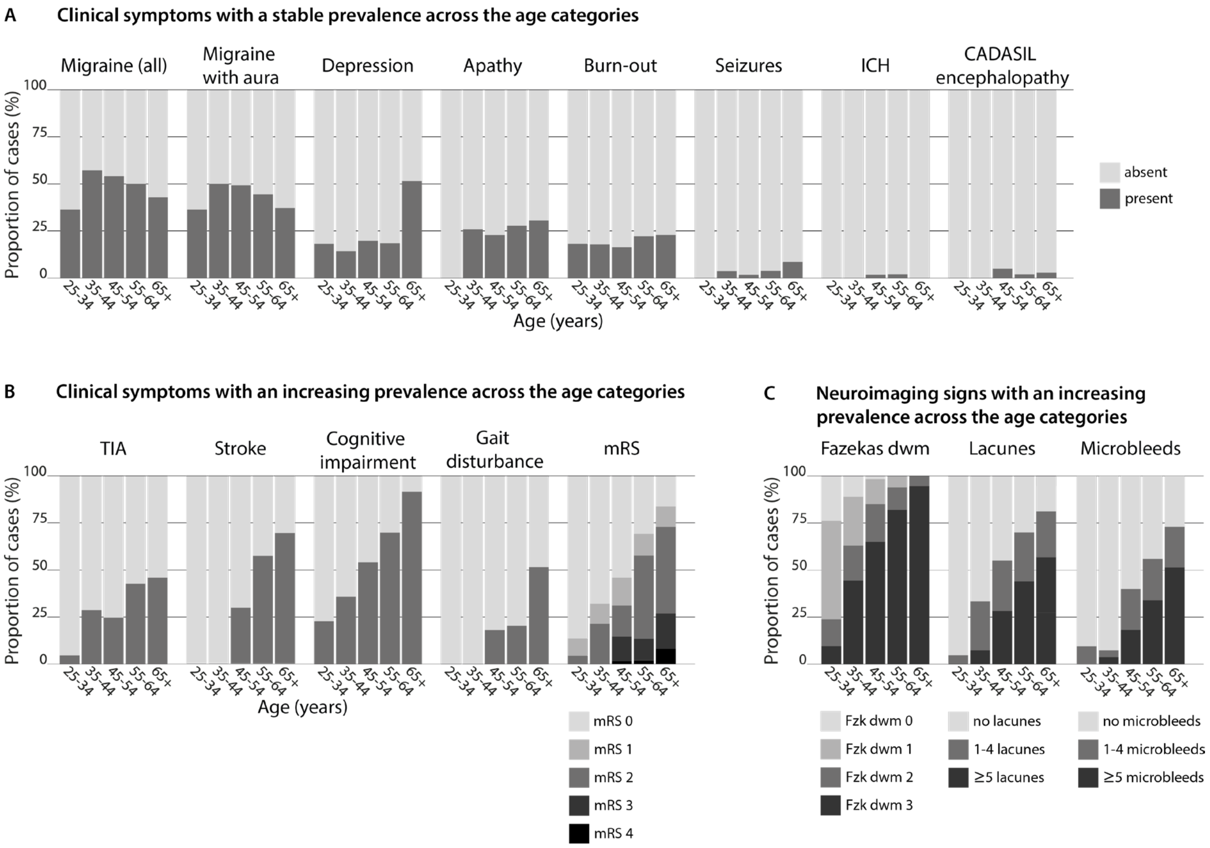

© 2024 Gravesteijn G et al. *JAMA Neurology.*

eFigure 2: Sequentiality of the NOTCH3-SVD Staging System in the Discovery and Validation Cohorts

A case is considered to be a sequential case if the case fulfills the criterion for a certain stage, as well as for all lower stages. (A) The number of sequential cases was 95.3% in the discovery cohort and 93.6% in the validation cohorts. (B) Per disease stage, the criteria that are fulfilled (full box) and not fulfilled (empty box) are shown, as well as the patient count per disease stage for all sequential cases. (C) All non-sequential cases in the discovery and validation cohorts are shown per combination of criteria which are fulfilled. The most prevalent non-sequential cases are individuals who have Fzk dwm 1 in combination with 1-4 lacunes or ≥5 lacunes (n=44 and n=18, respectively), or cases with 1-4 lacunes with mRS 3 or higher (n=23, n=14 and n=1).

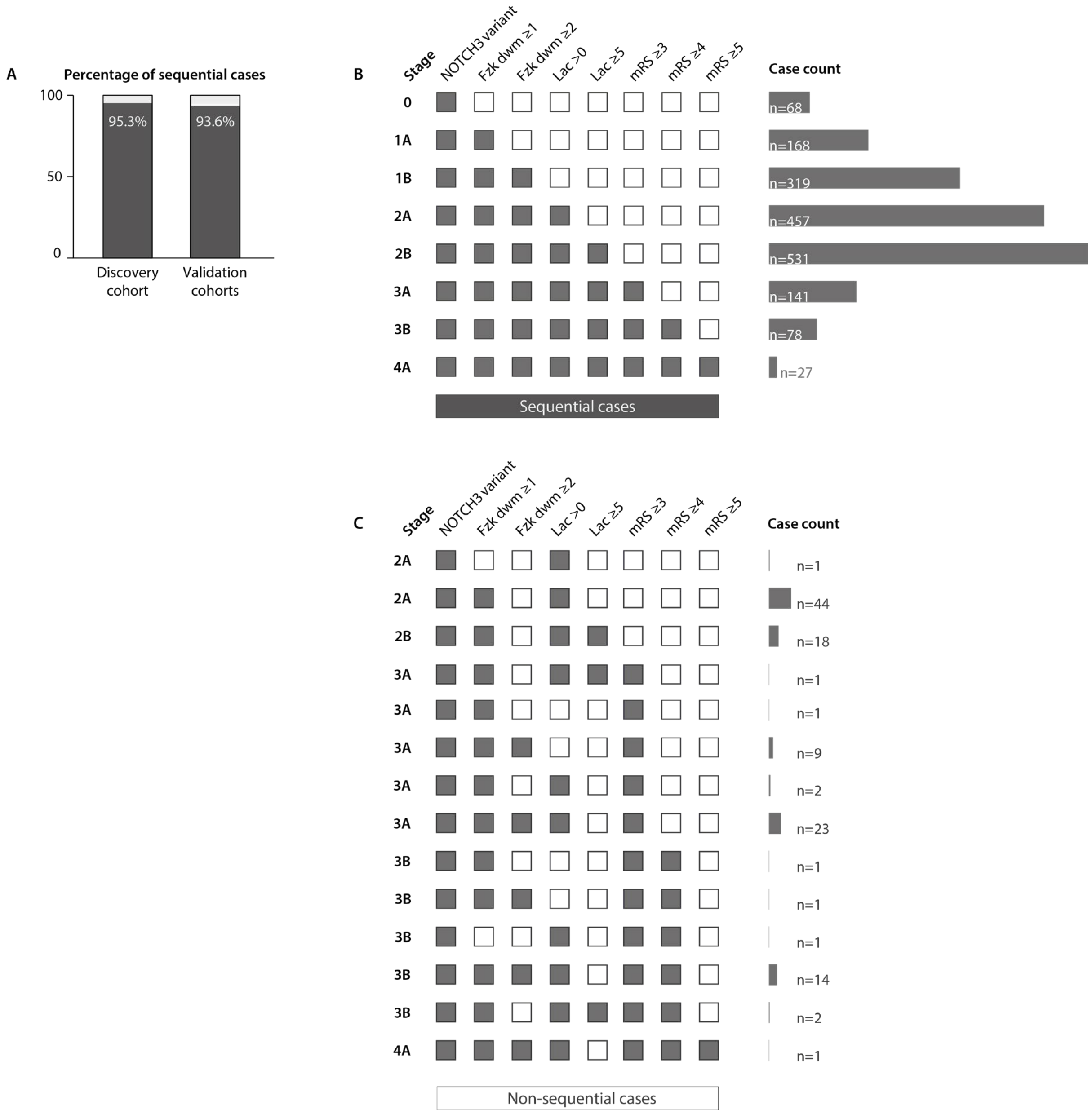

© 2024 Gravesteijn G et al. *JAMA Neurology.*

eFigure 3: Distribution of CADASIL Features per NOTCH3-SVD Stage

The prevalence of the presence of the depicted feature per NOTCH3-SVD stage for (A) clinical features and (B) neuroimaging features not included in the NOTCH3-SVD staging system itself. NOTCH3-SVD stage 4A was not included as there were no cases in this stage in the discovery cohort.

A Clinical features across NOTCH3-SVD stages in the discovery cohort (Leiden DiViNAS cohort)

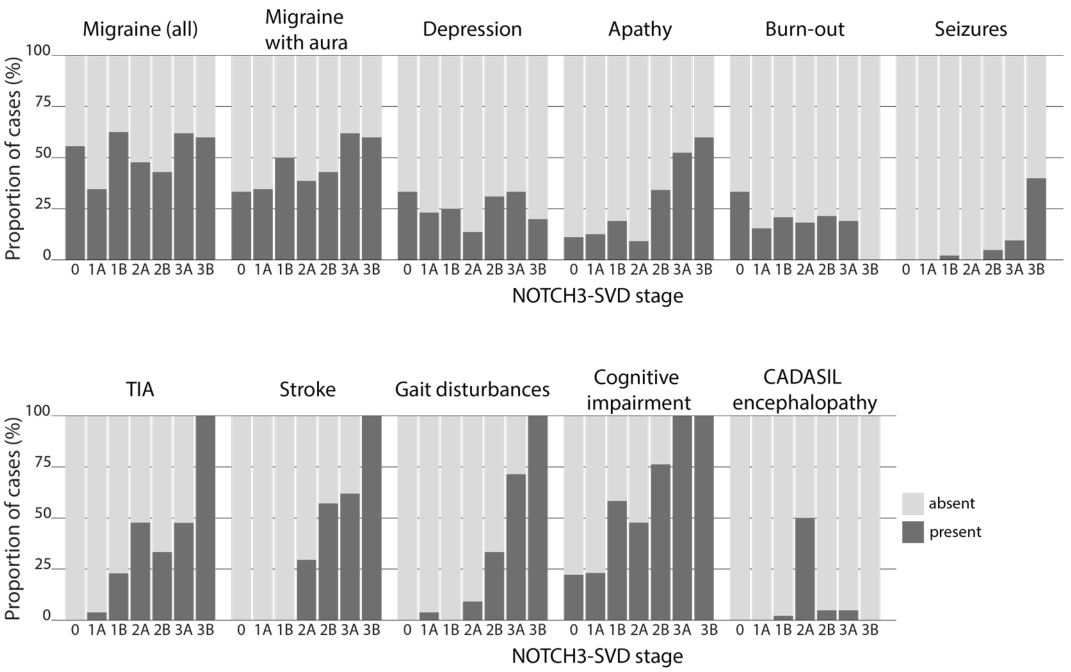

B Neuroimaging features across NOTCH3-SVD stages in the discovery cohort (Leiden DiViNAS cohort)

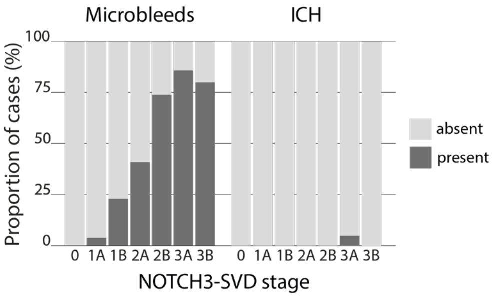

© 2024 Gravesteijn G et al. *JAMA Neurology.*

eFigure 4: Graphical Comparison of the NOTCH3-SVD Staging System With the Previously Reported Anisetti System

The distribution of sequential and non-sequential cases is shown for patients in the DiViNAS study when applying the CADASIL grading scale as suggested by Anisetti et al (A,B), and when applying the NOTCH3-SVD staging system (C,D). The NOTCH3-SVD staging system provided a better resolution in separating different disease stages (D) and had a higher percentage of sequential cases (E). The NOTCH3-SVD staging system was especially able to provide an increased resolution for individuals assigned to stage 2 of the Anisetti-system.

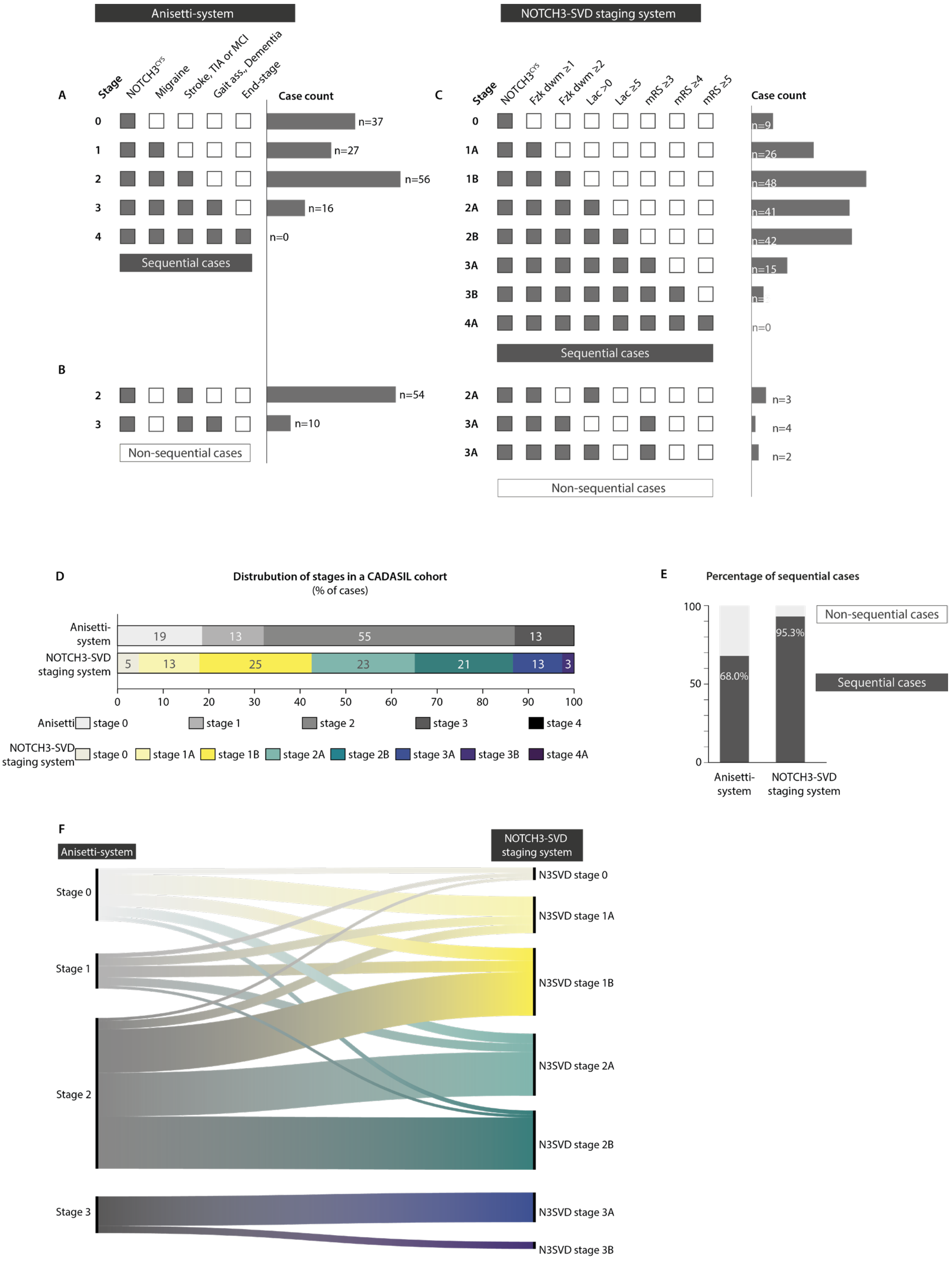

© 2024 Gravesteijn G et al. *JAMA Neurology.*

eFigure 5: Boxplots Showing the Association Between the NOTCH3-SVD Staging System and Age, Global Cognition, Processing Speed, BPF, PSMD and NfL per Cohort

eFigure 5A: Association between NOTCH3-SVD stages and age per cohort

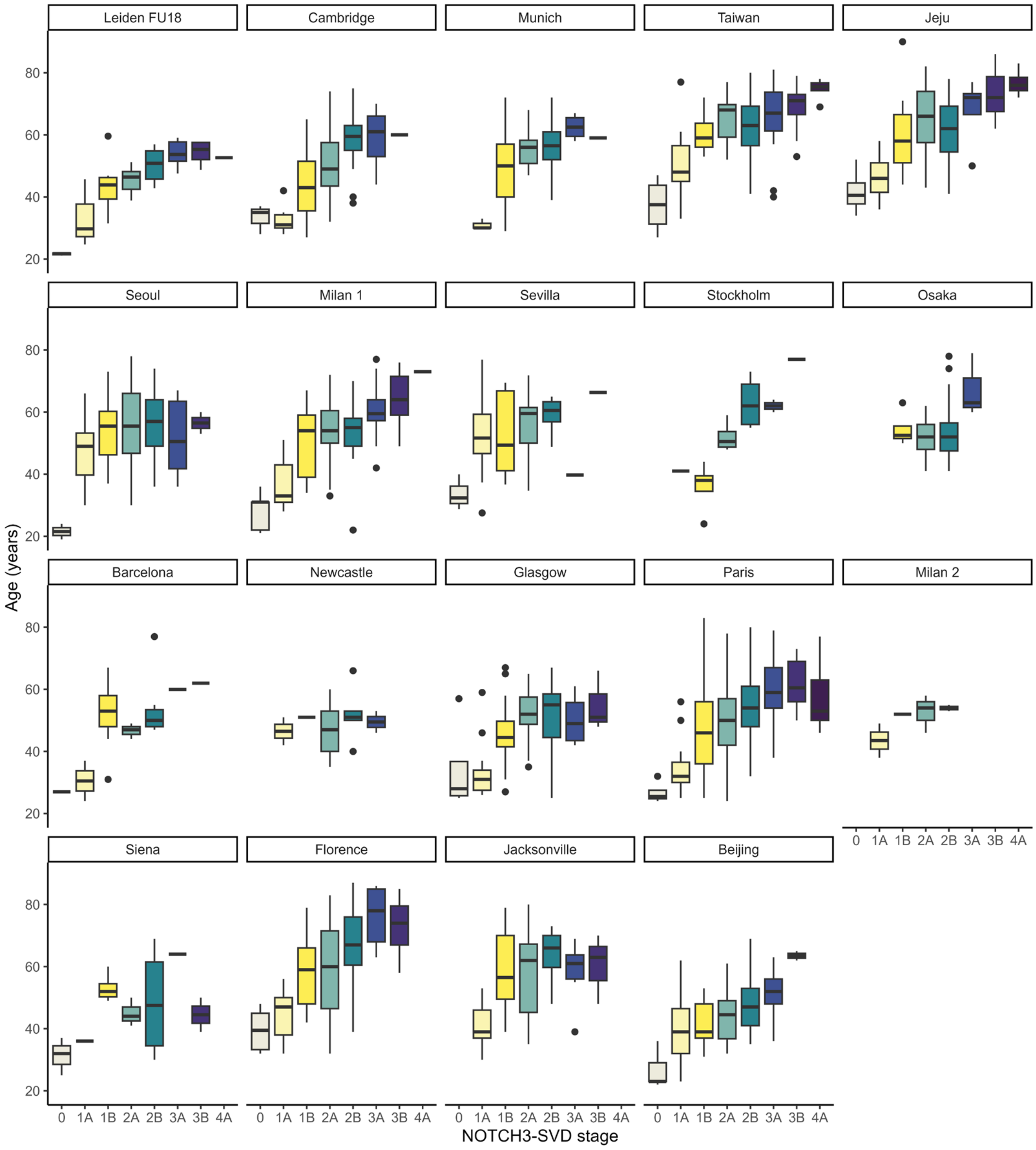

© 2024 Gravesteijn G et al. *JAMA Neurology.*

eFigure 5B: Association between NOTCH3-SVD stages and global cognition per cohort

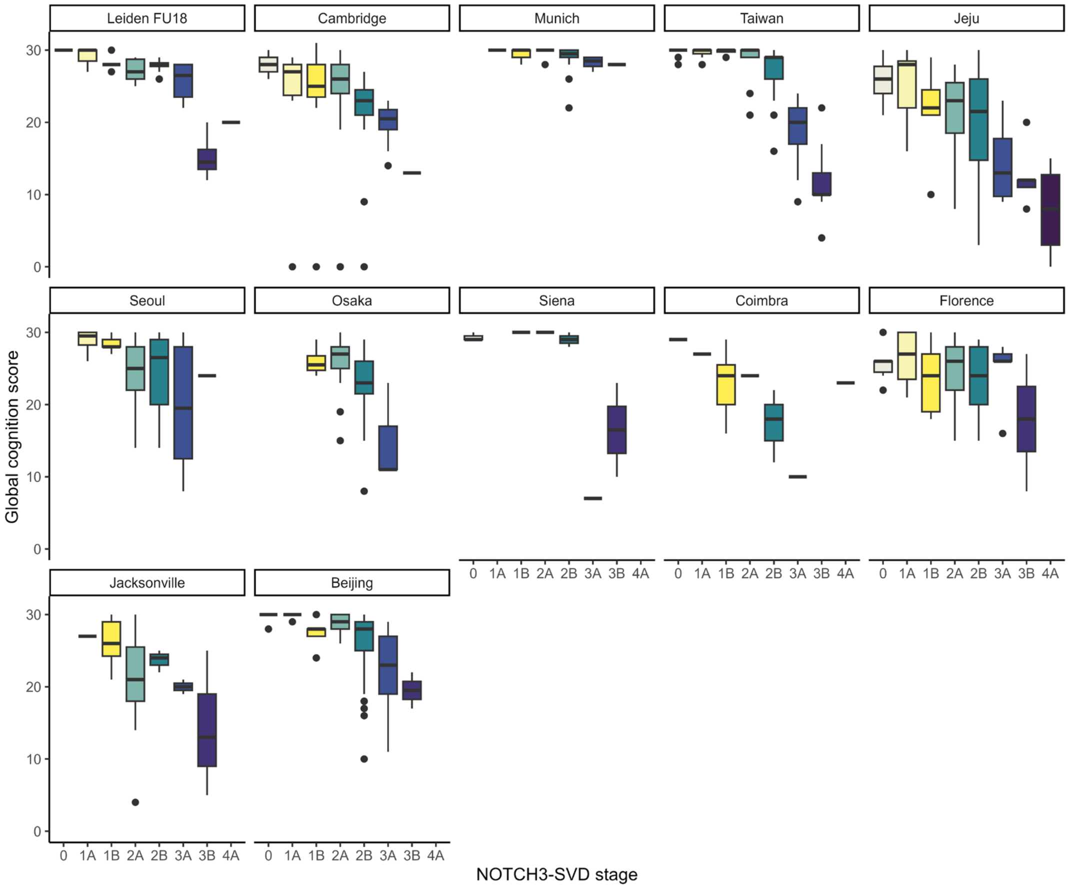

eFigure 5C: Association between NOTCH3-SVD stages and processing speed per cohort

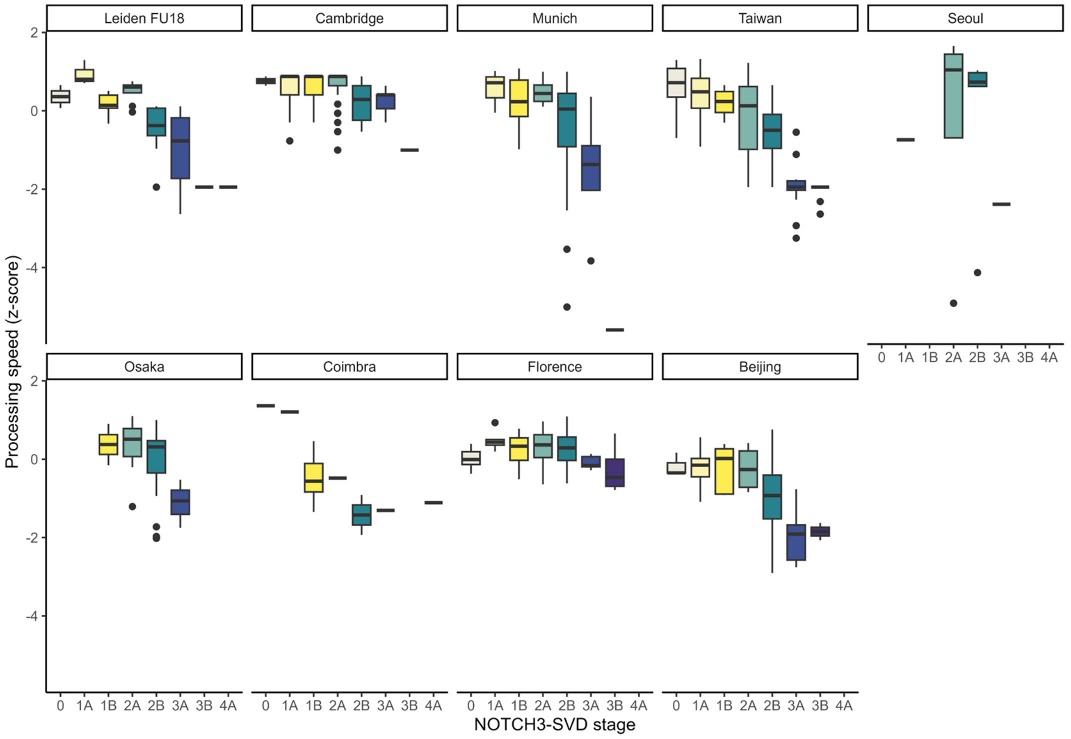

© 2024 Gravesteijn G et al. *JAMA Neurology.*

eFigure 5D: Association between NOTCH3-SVD stages and BPF per cohort

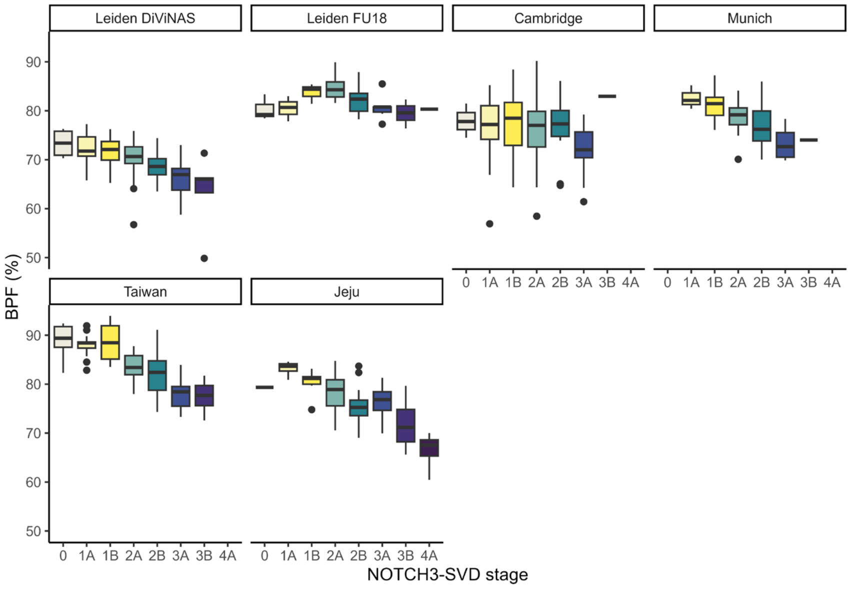

eFigure 5E: Association between NOTCH3-SVD stages and PSMD per cohort

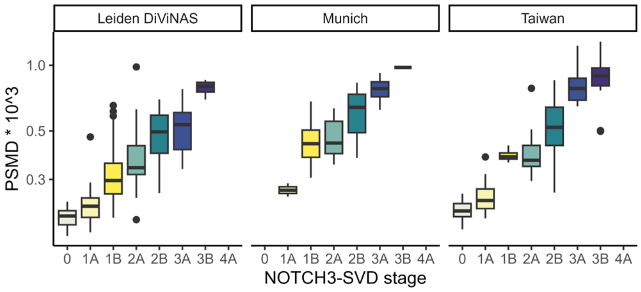

eFigure 5F: Association between NOTCH3-SVD stages and NfL per cohort

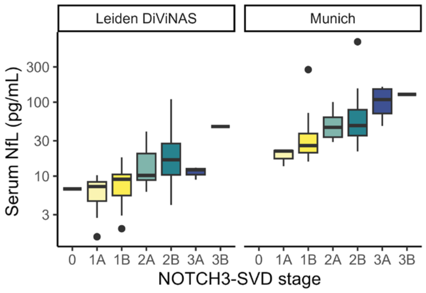

© 2024 Gravesteijn G et al. *JAMA Neurology.*

eFigure 6: Distribution of NOTCH3-SVD Stages by Sex

The distribution of NOTCH3-SVD stages is shown by sex based on data from all cohorts. Males are on average classified in a higher disease stage than females, also after correcting for age (Chi-square test $\chi^2 = 141$, $df = 7$, $P<.001$, Ordinal regression $\chi^2 = 605$, $P_{\text{adjusted for age}} < .001$).

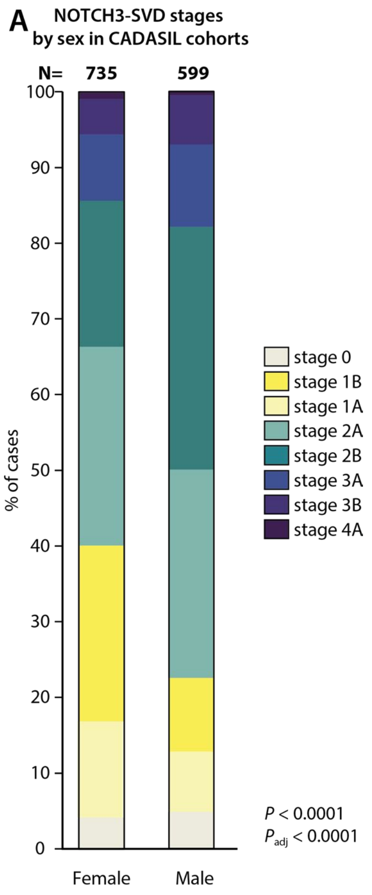

© 2024 Gravesteijn G et al. *JAMA Neurology.*
