# Supplementary material for: Disease Severity Staging System for NOTCH3-Associated Small Vessel Disease, Including CADASIL
Source: JAMA Neurol. 2024 Nov 29;82(1):49–60. doi: 10.1001/jamaneurol.2024.4487 (PMC13340440; doi:10.1001/jamaneurol.2024.4487)
Supplement: Supplement 2. — Data Sharing Statement [file jamaneurol-e244487-s002.pdf]

Data Sharing Statement

Gravesteijn. Disease Severity Staging System for NOTCH3-Associated Small Vessel Disease, Including CADASIL. *JAMA Neurol*. Published November 29, 2024.
doi:10.1001/jamaneurol.2024.4487

Data
Data available: No

Additional Information
Explanation for why data not available: The anonymized patient dataset of the respective research sites can be shared with qualified investigators upon reasonable request to the principle investigator of that specific research site. The online tool to perform disease staging is freely available at www.lumc.nl/notch3-svd-staging-system.
